# Targeting p21‐Positive Senescent Chondrocytes via IL‐6R/JAK2 Inhibition to Alleviate Osteoarthritis

**DOI:** 10.1002/advs.202410795

**Published:** 2025-01-23

**Authors:** Xiang Zhao, Jieming Lin, Feng Liu, Yu Zhang, Bo Shi, Chunhui Ma, Ziqi Wang, Song Xue, Qingrong Xu, Hongda Shao, Jingxing Yang, Yanzheng Gao

**Affiliations:** ^1^ Department of Surgery of Spine and Spinal Cord Henan Provincial People's Hospital, People's Hospital of Zhengzhou University People's Hospital of Henan University No.7 Weiwu Road Zhengzhou Henan 450003 China; ^2^ Department of Orthopaedics Renji Hospital School of Medicine Shanghai Jiao Tong University No. 160 Pujian Road Shanghai 200127 China; ^3^ Department of Interventional Oncology Renji Hospital School of Medicine Shanghai Jiao Tong University 160 Pujian Road Shanghai 200127 China; ^4^ Division of Spine Surgery Department of Orthopedic Surgery Affiliated Drum Tower Hospital Medical School of Nanjing University Nanjing 210008 China; ^5^ Department of Orthopedic Surgery Shanghai General Hospital Shanghai Jiao Tong University Shanghai 200080 China; ^6^ Department of Respiratory and Critical Care Medicine Zhengzhou University People's Hospital Henan Provincial People's Hospital Zhengzhou 450003 China; ^7^ Clinical Research Centre Zhujiang Hospital Southern Medical University Guangzhou Guangdong 510000 China; ^8^ Department of Nuclear Medicine Ren Ji Hospital Shanghai Jiaotong University School of Medicine 160 Pujian Road Shanghai 200127 China; ^9^ School of Biomedical Engineering Med‐X Research Institute Shanghai Jiao Tong University Shanghai 200030 China

**Keywords:** cartilage degeneration, interleukin‐6 receptor, JAK2 signaling, osteoarthritis, senescent cells

## Abstract

Osteoarthritis (OA) is an age‐related degenerative joint disease, prominently influenced by the pro‐inflammatory cytokine interleukin‐6 (IL‐6). Although elevated IL‐6 levels in joint fluid are well‐documented, the uneven cartilage degeneration observed in knee OA patients suggests additional underlying mechanisms. This study investigates the role of interleukin‐6 receptor (IL‐6R) in mediating IL‐6 signaling and its contribution to OA progression. Here, significantly elevated IL‐6R expression is identified in degenerated cartilage of OA patients. Further, in vivo experiments reveal that intra‐articular injection of recombinant IL‐6R protein or activation of gp130 (Y757F mutation) accelerates OA progression. Conversely, knockout of IL‐6R or JAK2, as well as treatment with a JAK inhibitor, alleviates OA symptoms. Mechanistically, chondrocytes derived from degenerative cartilage exhibit impaired nuclear localization of SOX9, a key regulator of cartilage homeostasis. JAK inhibition stabilizes SIRT1, reduces SOX9 acetylation, and thereby facilitates SOX9 nuclear localization, promoting cartilage repair. Additionally, the JAK inhibitor‐induced apoptosis in p21‐positive senescent cells, and their targeted clearance successfully alleviates OA in p21‐3MR mice. In conclusion, these findings reveal a novel mechanism by which inhibiting the IL‐6R/JAK2 pathway can alleviate OA. Furthermore, this study proposes targeting p21‐positive senescent cells as a new therapeutic strategy for OA.

## Graphical abstract

This study uncovers a novel mechanism behind uneven cartilage degeneration in knee osteoarthritis (OA). Elevated IL‐6R expression activates the IL‐6 pathway, leading to SIRT1 degradation, chondrocyte senescence, and reduced SOX9 activity, which impairs cartilage matrix synthesis. Notably, the JAK inhibitor tofacitinib prevents IL‐6R‐mediated senescence, and targeted clearance of p21‐positive senescent chondrocytes significantly alleviates OA symptoms, offering a promising therapeutic strategy.

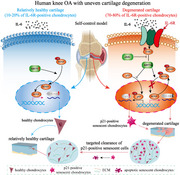

## 1 Introduction

Osteoarthritis (OA) is a prevalent degenerative joint disease that affects millions globally, marked by progressive cartilage degradation, pain, and reduced mobility.^[^ ^1^ ^]^ Despite its prevalence, the exact etiology of OA remains unclear, and current clinical strategies are often insufficient for managing the condition in many patients.^[^ ^2^ ^]^ With the aging population and the increasing burden of OA,^[^ ^3^ ^]^ there is an urgent need to find effective management and treatment options.^[^ ^4^ ^]^ Chronic low‐grade inflammation has been recognized as a key driver in the progression of OA.^[^ ^5^ ^]^ In recent years, anti‐inflammatory therapies have shown promise in alleviating symptoms and slowing disease progression in some OA patients.^[^ ^6^ ^]^ However, the limited efficacy of these existing therapies is mainly due to an incomplete understanding of their underlying mechanisms.^[^ ^7^ ^]^ Therefore, a deeper comprehension of these mechanisms is essential for developing more effective OA therapies.

Chronic inflammation is a critical factor in the imbalance between catabolic and anabolic activities in OA.^[^ ^8^ ^]^ This imbalance is primarily driven by various inflammatory mediators, which enhance catabolic activities and accelerate disease progression.^[^ ^9^ ^]^ Among these mediators, interleukin‐6 (IL‐6) is particularly notable as a pro‐inflammatory cytokine with a significant role in OA progression, making it a focal point of extensive research.^[^ ^10^ ^]^ Previous studies have demonstrated that IL‐6 levels were markedly elevated in the synovial fluid, synovium, and cartilage of patients with knee OA, exacerbating disease severity and intensifying joint pain and inflammation.^[^ ^10^, ^11^ ^]^ IL‐6 exerted its effects by binding to its receptor IL‐6R, thereby activating the gp130/JAK2 signaling pathway, which lead to the excessive production of matrix metalloproteinases (MMPs). In return, the increased activity of these enzymes accelerated the degradation of the cartilage matrix, further driving the pathological progression of OA.^[^ ^12^ ^]^ Given that IL‐6R plays a central role in mediating the effects of IL‐6, its expression levels in cartilage may directly influence the severity of degeneration. Interestingly, studies have shown that cartilage degeneration is unevenly distributed among most knee OA patients, likely due to the varying severity of the degenerative process across different regions.^[^ ^13^ ^]^ To better understand this regional disparity, recent research has used the relatively healthy lateral cartilage as a control group while designating the severely worn medial cartilage as the degeneration group.^[^ ^14^ ^]^ However, the elevated IL‐6 levels in the joint fluid did not fully account for the more severe degeneration of the medial cartilage. It remains unclear whether changes in IL‐6R expression contribute to this imbalance. Therefore, we hypothesize that degenerated cartilage may exhibit higher IL‐6R expression, which could participate in OA progression through JAK2/STAT3 activation. Investigating the precise impact of increased IL‐6R expression in OA cartilage, as well as its potential value as a therapeutic target, remains a crucial and challenging research area.

IL‐6 is a critical mediator in OA, not only driving cartilage degradation but also influencing repair mechanisms, which adds complexity to its role in disease progression. This cytokine plays a dual role in cartilage metabolism, affecting both catabolic and anabolic processes. Chronic low‐grade inflammation, characterized by elevated IL‐6 levels, promotes chondrocyte senescence, reducing cartilage matrix synthesis and contributing to the imbalance between cartilage degradation and regeneration.^[^ ^14^, ^15^ ^]^ The presence of senescent chondrocytes, which produce less collagen and exhibit a senescence‐associated secretory phenotype (SASP), is a hallmark of OA.^[^ ^16^ ^]^ Current research is exploring IL‐6 pathway inhibitors, such as JAK inhibitors (JAKi), as potential “senotherapeutics” to address this imbalance by inhibiting the SASP.^[^ ^17^ ^]^ In the context of OA, inhibiting the JAK/STAT3 pathway has demonstrated the potential to slow cartilage degradation by reducing inflammation and the production of MMPs.^[^ ^12b,c^ ^]^ However, given the involvement of the JAK/STAT3/BCL2 pathway in cell survival,^[^ ^12^, ^18^ ^]^ it is not yet clear whether JAK inhibition could induce apoptosis in senescent cells. Hence, this study aims to clarify this aspect by examining the effects of JAK inhibitors on the viability of senescent cells, with the goal of determining whether targeting IL‐6R and JAK2 can effectively mitigate OA by promoting the clearance of these cells.

Therefore, the critical role of chronic low‐grade inflammation and cellular senescence in the progression of OA has increasingly garnered attention. IL‐6 and its downstream signaling pathways, particularly the JAK2/STAT3 axis, are recognized as key mediators of cartilage degradation and the pathological progression of OA. However, the precise mechanisms by which IL‐6R activation contributes to cellular senescence and matrix degradation remain incompletely understood. Recent studies suggest that senescent cells, characterized by markers such as p21, may exacerbate tissue inflammation and cartilage degeneration through the secretion of pro‐inflammatory mediators. Despite advances in understanding the IL‐6 signaling cascade, the therapeutic potential of selectively targeting senescent cells and modulating IL‐6R/JAK2 signaling has yet to be fully explored. This study aims to address this gap by elucidating the role of IL‐6R/JAK2 activation in OA pathogenesis and assessing its impact on senescent chondrocytes and cartilage homeostasis, thereby providing a theoretical foundation for novel therapeutic strategies.

## 2 Results

### 2.1 The High Expression of IL‐6R/p‐JAK2 Is Associated with More Severe Cartilage Degeneration in OA

**Figure 1:**
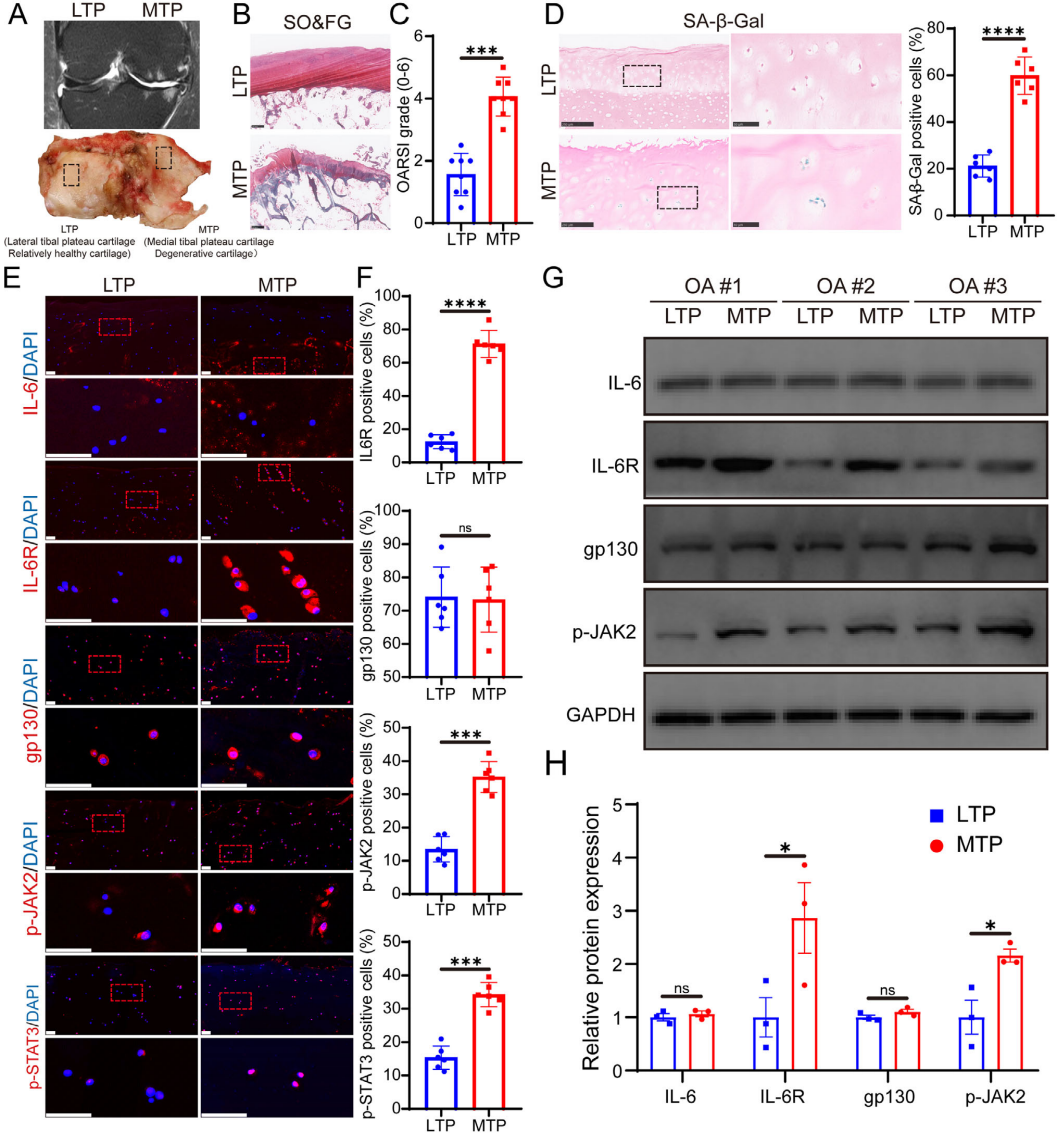
IL‐6R/p‐JAK2 expression is drastically increased in human OA degenerated cartilage. A) MRI (T2) images display the coronal view of the knee joints from OA patients prior to surgery. The articular cartilage samples were obtained during total knee replacement procedures. The black dotted boxes highlight the relatively healthy lateral tibial plateau (LTP) and the severely degenerated medial tibial plateau (MTP) areas. B) Safranin‐O/Fast Green (SO&FG) staining was performed on cartilage sections from both LTP and MTP regions. Scale bar: 500 µm. C) OARSI grading of the cartilage was conducted based on regions of interest (*n* = 8). D) Senescence‐associated β‐galactosidase (SA‐β‐Gal) staining was used to evaluate cell senescence in LTP and MTP cartilage (*n* = 6). Scale bar: 250 µm. E) The expression of IL‐6, IL‐6R, gp130, p‐JAK2, and p‐STAT3 in chondrocytes was evaluated by immunofluorescence staining in LTP or MTP cartilage. Scale bar: 50 µm. F) The proportion of IL‐6R, gp130, p‐JAK2, and p‐STAT3 positive cells was quantified by immunofluorescence (*n* = 6). G,H) Western blotting was performed to detect and quantify the protein levels of IL‐6, IL‐6R, gp130, and p‐JAK2 in cartilage samples from both MTP and LTP regions in three OA patients (*n* = 3). Abbreviations: LTP, medial side of the tibial plateau; MTP, lateral side of the tibial plateau; SO&FG, Safranin‐O/Fast Green; OARSI, Osteoarthritis Research Society International; gp130, glycoprotein 130; STAT3, Signal transducer and activator of transcription 3. Quantitative data are shown as mean ± S.D. Statistical significance was determined using a two‐sided paired Student's t‐test. **p < 0.01, ***p < 0.001, ****p < 0.0001, ns, no significant.

To investigate the role of IL‐6R in uneven cartilage degeneration, we established a self‐control model^[^ ^14b,c^ ^]^ by comparing the medial and lateral cartilage of the tibial plateau in knee OA patients (**Figure** **1**A). Detailed patient data is provided in Table S1 (Supporting Information). As anticipated, Safranin‐O Fast Green (SO&FG) staining revealed more severe degeneration in the medial tibial plateau (MTP) cartilage, while the lateral tibial plateau (LTP) cartilage remained relatively healthy (Figure 1B,C). Additionally, SA‐β‐Gal staining indicated a substantial presence of senescent cells in the degenerated cartilage (Figure 1D). Compared to the relatively healthy cartilage, the degenerated cartilage showed a significantly increased number of IL‐6R and p‐JAK2 positive cells, along with higher expression levels (Figure 1E–H). IL‐6R was also highly expressed in primary chondrocytes isolated from the degenerated cartilage (Figure S1, Supporting Information). However, there were no significant differences in the protein levels of IL‐6 and gp130 between the two groups (Figure 1G,H). Further tissue fluorescence staining revealed that IL‐6‐positive cells were scarcely detectable in both relatively healthy and degenerated cartilage, while a large number of gp130‐positive cells were present in both groups (Figure 1E,F). The mRNA levels of IL‐6R were significantly elevated in the degenerated cartilage tissue (Figure S2, Supporting Information), suggesting that endogenous IL‐6R is highly expressed in the degenerated cartilage. We also observed that IL‐6R expression significantly increased in the cartilage of both OA models in rats and mice compared to the sham group (Figure S3, Supporting Information). These findings suggest that high IL‐6R expression may play a critical role in activating the IL‐6 signaling pathway in degenerated cartilage in knee OA.

### 2.2 Intra‐Articular Injection of Recombinant IL‐6R Protein or gp130 Point Mutation Can Accelerate OA Progression

**Figure 2:**
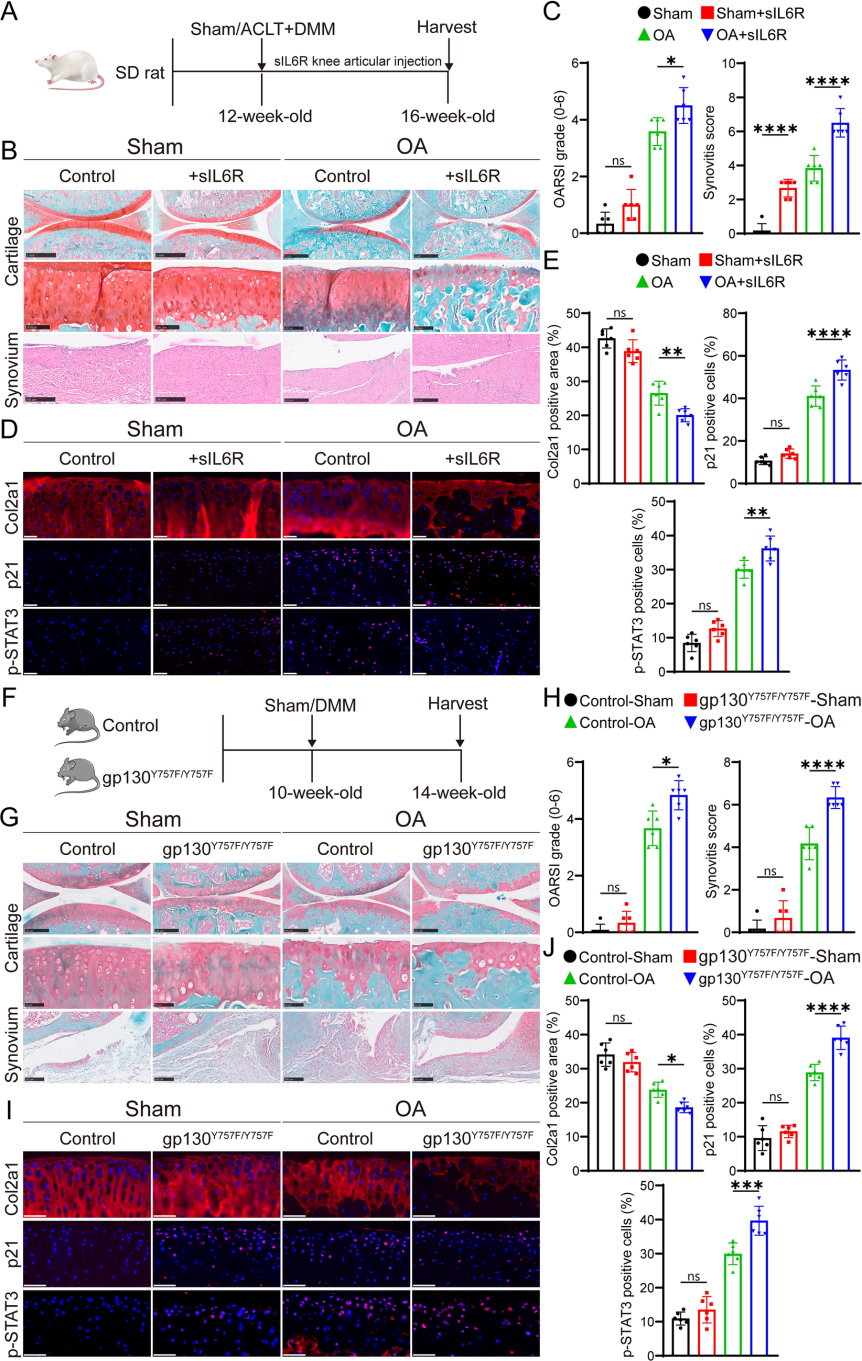
Intra‐articular administration of recombinant IL‐6R protein hastens the progression of OA in rats, while a gp130 point mutation accelerates OA development in mice. A) Diagram illustrating the intra‐articular injection of recombinant IL‐6R protein in a rat OA model. Rats underwent ACLT + DMM surgery or sham operation, followed by weekly injections of 50 µL IL‐6R protein (100 ng mL^−1^) for 4 weeks. Normal saline was used as a control (*n* = 6 rats per group). B) Safranin‐O/Fast Green (SO&FG) staining of cartilage and H&E staining of synovial tissue were performed 4 weeks post‐surgery. Scale bar: top:1 mm, middle: 100 µm, bottom: 250 µm. C) Quantification of OARSI and synovitis scores. D) Immunofluorescence analysis of Col2a1, p21, and p‐STAT3 expression in rat knee cartilage. Nuclei were counterstained with DAPI. Scale bar: 50 µm. E) Quantification of Col2a1‐positive area and the proportion of p21 and p‐STAT3‐positive cells by immunofluorescence for rat knee cartilage. F) Experimental schematic for evaluating the effects of gp130 mutations on OA progression in mice. G) SO&FG staining shows cartilage degradation and synovitis in gp130 mutant mice (gp130 ^Y757F/Y757F^) compared to wild‐type (WT) mice 4 weeks after DMM surgery or sham operation (*n* = 6 mice per group). Scale bar: top: 250 µm, middle: 50 µm, bottom: 100 µm. H) Quantification of OARSI and synovitis scores. I) Immunofluorescence staining of Col2a1, p21, and p‐STAT3 in mice knee cartilage. Nuclei were counterstained with DAPI. Scale bar: 50 µm. J) Quantitative analysis of Col2a1‐positive areas and the proportion of p21‐ and p‐STAT3‐positive cells in mouse knee cartilage. Abbreviations: ACLT, anterior cruciate ligament transection; DMM, destabilization of the medial meniscus; WT, wild type; SO&FG, Safranin‐O/Fast Green. Quantitative data are shown as mean ± S.D. One‐way ANOVA with Tukey's multiple comparisons was used for statistical analysis. *p < 0.05, **p < 0.01, ***p < 0.001, ****p < 0.0001, ns, no significant.

While previous research has shown increased sIL‐6R expression in OA synovial fluid,^[^ ^19^ ^]^ its role in disease progression remains unclear. In this study, we next explored the consequences of elevated IL‐6R levels by administering 50 µL of recombinant rat sIL‐6R (100 ng mL^−1^) into the knee joints of rats weekly (**Figure** **2**A). After one month, we found that sIL‐6R injection alone did not promote cartilage degeneration (Figure 2B,C). However, at this dosage, sIL‐6R significantly accelerated OA progression in the ACLT + DMM‐induced model (Figure 2B–E). Since IL‐6R relies on gp130 as its signal transducer,^[^ ^12a^ ^]^ we specifically activated gp130 through a point mutation (Y757F) to rule out any dosage‐related effects of sIL‐6R injection (Figure S4, Supporting Information). Our results indicated that 14‐week‐old mice carrying the gp130 (Y757F) mutation did not exhibit cartilage degeneration under sham surgery (Figure 2F–H). However, when subjected to the DMM surgery‐induced OA model, these mice demonstrated a marked increase in senescent chondrocytes and a more rapid progression of OA (Figure 2G–J). These findings suggest that the upregulation of IL‐6R expression plays a critical role in the progression of OA.

### 2.3 IL‐6R Gene Knockout Reduces Cartilage Degradation in Mice with OA

**Figure 3:**
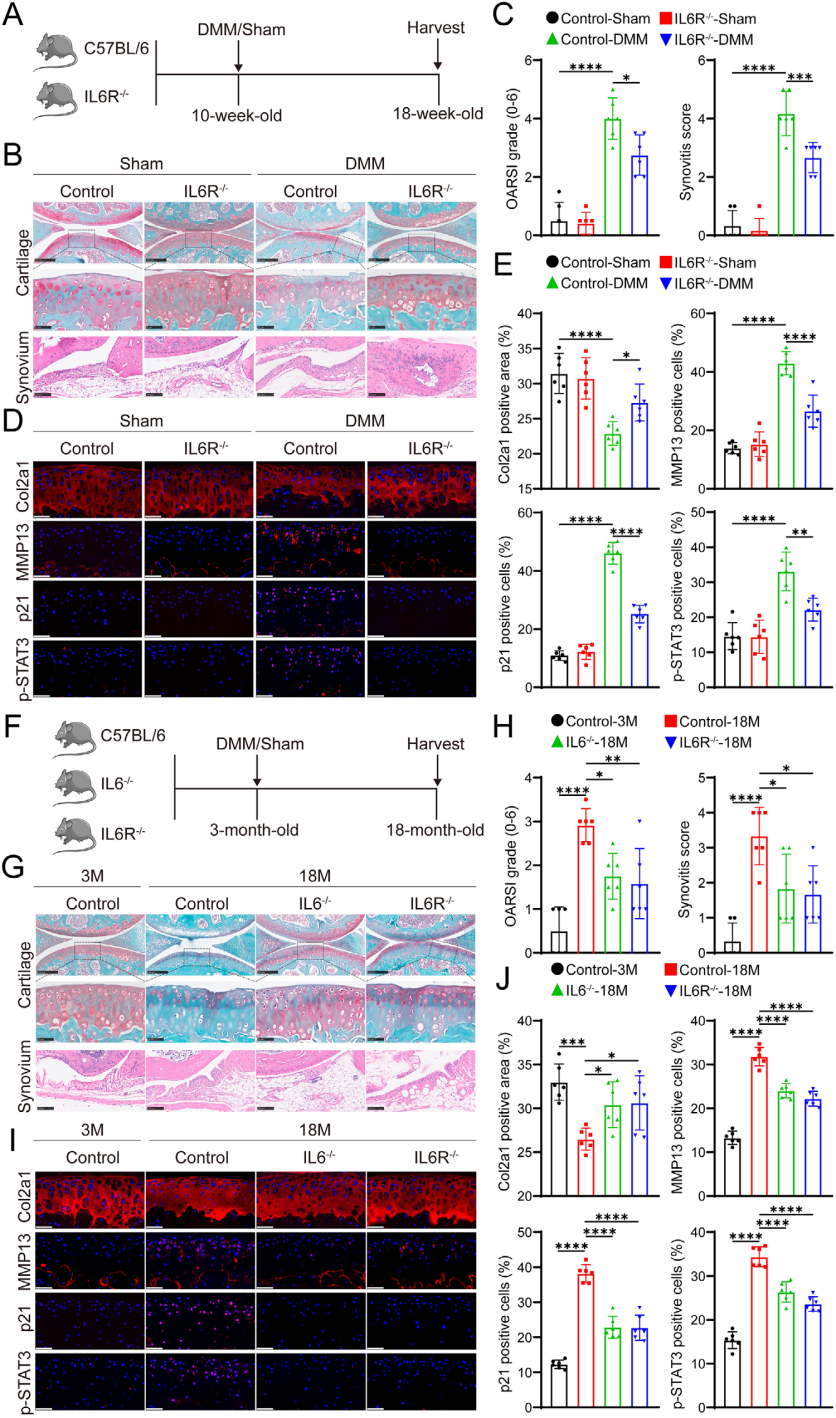
IL‐6R gene knockout reduces cartilage degradation in mice with OA induced by DMM surgery or aged mice. A) Schematic diagram illustrating the experimental setup for assessing IL‐6R KO alleviate mice OA induced by DMM. B) Cartilage staining with SO&FG and synovial tissue staining with H&E at 8 weeks post‐surgery. (*n* = 6 mice per group). Scale bar: top:250 µm, middle: 50 µm, bottom: 100 µm. C) Quantification of OARSI and synovitis scores. D) Immunofluorescence staining was conducted to evaluate Col2a1, MMP13, p21, and p‐STAT3 expression in mouse knee cartilage, with nuclei counterstained using DAPI. Scale bar: 50 µm. E) Quantitative analysis of Col2a1‐positive areas and the proportion of p21‐ and p‐STAT3‐positive cells in mouse knee cartilage using immunofluorescence. F) Schematic diagram showing the experimental setup for evaluating the effects of IL‐6 and IL‐6R gene knockout on knee joint homeostasis in aged mice. Wild‐type (WT) mice, IL‐6 KO mice, and IL‐6R KO mice were maintained under standard feeding conditions until 18 months of age. G) SO&FG staining of knee cartilage and H&E staining of synovial tissue were performed on knee samples from 3‐month‐old and 18‐month‐old WT, IL‐6 KO, and IL‐6R KO mice (*n* = 6 mice per group).Scale bar: top: 250 µm, middle: 50 µm, bottom: 100 µm. H) Quantification of OARSI and synovitis scores. I) Immunofluorescence staining assessed the expression of Col2a1, MMP13, p21, and p‐STAT3 in mouse knee cartilage, with nuclei counterstained using DAPI. Scale bar: 50 µm. J) Quantitative analysis of Col2a1‐positive areas and the proportion of MMP13‐, p21‐, and p‐STAT3‐positive cells in mouse knee cartilage using immunofluorescence. Quantitative data are shown as mean ± S.D. Statistical significance was determined using one‐way ANOVA with Tukey's multiple comparisons: *p < 0.05, **p < 0.01, ***p < 0.001, ****p < 0.0001.

Next, to elucidate the role of IL‐6R in mitigating OA, we used a transgenic mouse model with IL‐6R gene KO (Figure S5, Supporting Information). At 10 weeks of age, the mice underwent DMM surgery (**Figure** **3**A). SO&FG staining revealed that IL‐6R KO significantly slowed the progression of OA and reduced synovial hyperplasia (Figure 3B,C). Immunofluorescence analysis showed that, compared to wild‐type (WT) mice, the activation of the JAK2‐STAT3 pathway and the number of p21‐positive senescent cells were significantly reduced in the IL‐6R KO group after DMM surgery (Figure 3D,E; Figure S6, Supporting Information). This finding aligns with results from IL‐6 KO mice, where a significant reduction in p21‐positive senescent cells was also observed in the OA model induced by DMM surgery (Figures S7, S8, Supporting Information). We further assessed the effects of IL‐6R KO on the articular cartilage of aged mice (Figure 3F). In aged WT mice, mild cartilage degeneration and an increase in p21‐positive cells were noted (Figure 3G,H). In contrast, both IL‐6 and IL‐6R KO significantly delayed cartilage degeneration and decreased the number of p21‐positive senescent cells in the aged group (Figure 3G–J). These results suggest that targeting IL‐6R is a pivotal strategy for treating OA.

### 2.4 Inducible KO of the JAK2 Gene Reduces Cartilage Degradation in Mice with OA

**Figure 4:**
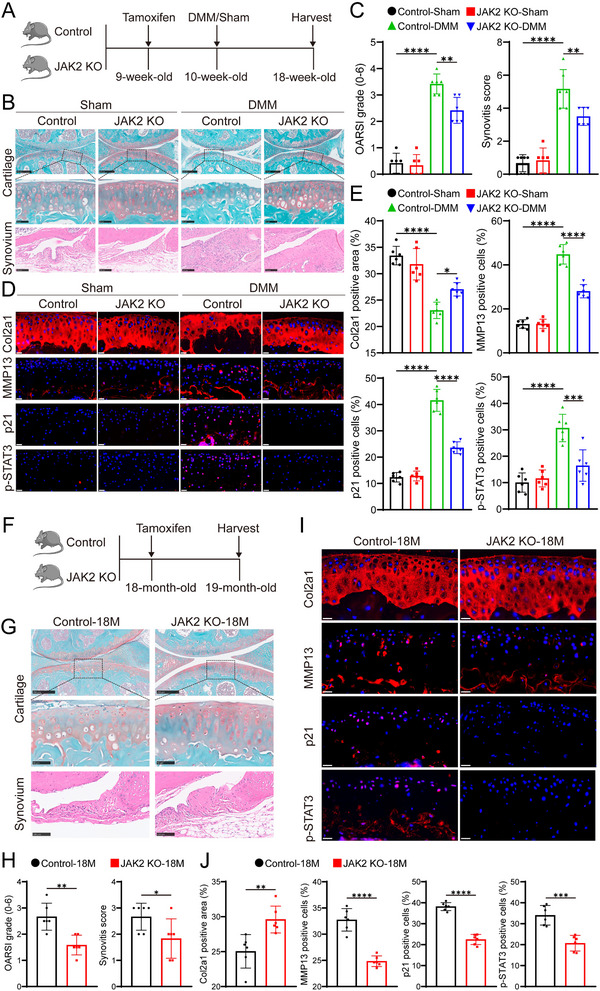
Inducible KO JAK2 gene mitigates cartilage degradation in mice with OA, either induced by DMM surgery or in aged mice. A) Schematic representation of the experimental design used to assess the effects of inducible JAK2 knockout on OA progression in mice following DMM surgery. B) Assessment of cartilage degradation and synovial inflammation in JAK2 KO and JAK2^flox/flox^ mice 8 weeks post‐surgery, utilizing Safranin‐O/Fast Green (SO&FG) and H&E staining (*n* = 6 mice per group). Scale bar: top: 250 µm, middle: 50 µm, bottom: 100 µm. C) Quantification of OARSI and synovitis scores. D) Immunofluorescence staining of Col2a1, MMP13, p21, and p‐STAT3 in mice knee cartilage, with DAPI‐stained nuclei. Scale bar: 20 µm. E) Analysis of Col2a1‐positive area and the proportion of MMP13, p21, and p‐STAT3‐positive cells. F) Schematic diagram showing the experimental setup for evaluating the effects of JAK2 KO on cartilage homeostasis in 18‐month‐old mice. G) SO&FG staining of knee cartilage and H&E staining of synovial tissue were performed on knee samples from 19‐month‐old JAK2 KO and JAK2^flox/flox^ mice (*n* = 6 mice per group). Scale bar: top:250 µm, middle: 50 µm, bottom: 100 µm. H) Quantification of OARSI and synovitis scores. I) Immunofluorescence staining assessed the expression of Col2a1, MMP13, p21, and p‐STAT3 in mouse knee cartilage, with nuclei counterstained using DAPI. Scale bar: 20 µm. J) Quantitative analysis of Col2a1‐positive areas and the proportion of MMP13‐, p21‐, and p‐STAT3‐positive cells in mouse knee cartilage. Quantitative data are presented as mean ± S.D. Statistical significance in (C) and (E) was determined using one‐way ANOVA with Tukey's multiple comparisons, while in (H) and (J) a two‐sided unpaired Student's t‐test was used: *p < 0.05, **p < 0.01, ***p < 0.001, ****p < 0.0001, ns, not significant.

The JAK2/STAT3 pathway is a critical component of the IL‐6 signaling cascade,^[^ ^12c^ ^]^ yet its role in alleviating OA through specific JAK2 inhibition has been underexplored. In this study, we investigated the impact of conditionally KO the JAK2 gene on OA progression (**Figure** **4**A; Figure S9, Supporting Information). Our findings demonstrated that conditional JAK2 KO significantly reduced the number of p21‐positive senescent cells in the knee's articular cartilage, effectively slowing OA progression in the DMM model (Figure 4B–E). In aged mice, JAK2 KO also decreased the number of senescent chondrocytes and alleviated cartilage degeneration in the knee joint (Figure 4F,G). These results suggest that targeting the IL‐6R/JAK2 pathway could be a promising therapeutic strategy for mitigating OA progression.

### 2.5 Tofacitinib, a JAK Inhibitor (JAKi), Alleviates OA Progression

**Figure 5:**
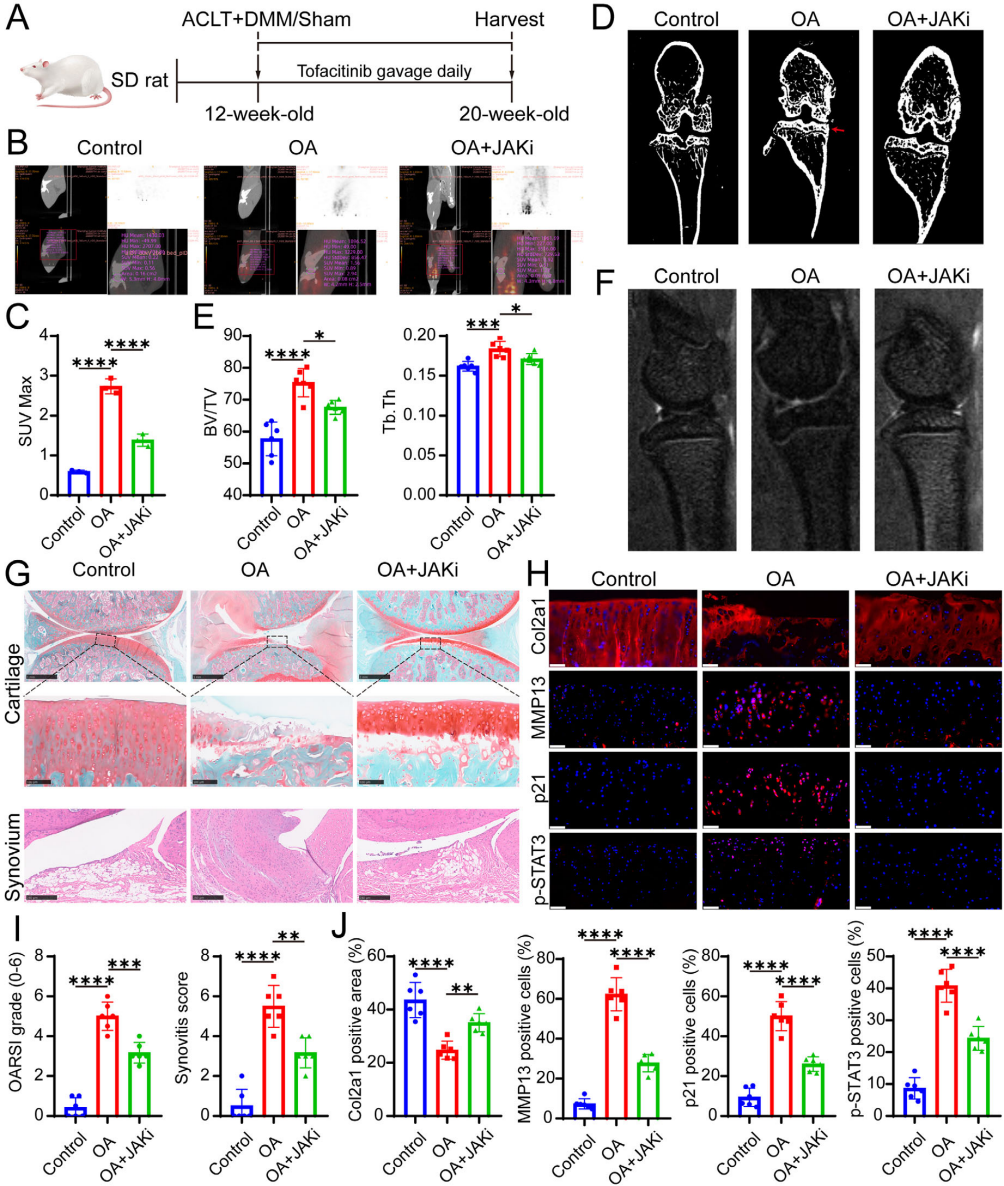
JAKi treatment alleviates OA in rats induced by ACLT + DMM surgery. A) Schematic diagram outlining the experimental protocol for assessing the effects of JAK inhibitor (JAKi) treatment on OA progression in rats post‐ACLT + DMM surgery. Rats were administered either vehicle (Veh) or JAKi (tofacitinib) via daily gavage, starting 3 days after surgery and continuing for 7 weeks. B,C) Eight weeks post‐surgery, rats received an ^18^F‐FDG injection through the tail vein, followed by PET/CT scans targeting the knee joint. Representative images are shown. The maximum standardized uptake value (SUVmax) at the knee joint was used to evaluate the treatment effects (*n* = 3 rats per group). D,E) Subchondral bone volume/tissue volume (BV/TV, %) and trabecular thickness (Tb. Th, mm) were analyzed by microtomography in sham, OA, and OA + JAKi rats. Quantitative results are provided. F) Representative MRIs (T2) of knee articular in sham, OA, and OA + JAKi rats. G) Cartilage damage and synovitis were evaluated at 8 weeks using SO&FG and H&E staining. Scale bar: top:1 mm, middle: 100 µm, bottom: 250 µm. H) Immunofluorescence staining was conducted to evaluate the expression of Col2a1, MMP13, p21, and p‐STAT3 in rat knee cartilage, with DAPI‐stained nuclei (*n* = 6 rats per group). Scale bar: 50 µm. I) Quantification of OARSI and synovitis scores. J) Analysis of Col2a1‐positive area and the proportion of MMP13‐, p21‐, and p‐STAT3‐positive cells in rat knee cartilage. Abbreviations: JAKi, JAK inhibitors; Veh, vehicle. Quantitative data are shown as mean ± S.D. One‐way ANOVA with Tukey's multiple comparisons was used for statistical analysis. *p < 0.05, **p < 0.01, ***p < 0.001, ****p < 0.0001, ns, no significant.

To assess the clinical potential of JAKi in OA, we next evaluated the efficacy of tofacitinib, a JAK inhibitor, in preventing cartilage degeneration (**Figure** **5**A). Currently, there is a paucity of approved disease‐modifying drugs specifically for improving OA outcomes.^[^ ^7a^ ^]^ However, JAKi, such as tofacitinib, is clinically used to treat rheumatoid arthritis (RA) by effectively reducing synovial inflammation and preventing RA‐induced cartilage erosion.^[^ ^20^ ^]^ In human degenerated cartilage, the number of p‐JAK1 and p‐JAK3 positive cells was significantly increased compared to relatively healthy cartilage (Figure S10, Supporting Information). JAKi treatment suppressed the uptake of ^18^F‐FDG in the knee joints of the OA group, indicating a reduction in inflammation (Figure 5B,C). Additionally, JAKi treatment alleviated subchondral bone remodeling in the OA group (Figure 5D,E). The results showed that JAKi significantly mitigated cartilage degeneration in OA models in rats and markedly reduced the number of p21‐positive senescent cells (Figure 5F–J; Figure S11, Supporting Information). After 8 weeks of treatment, safety evaluations revealed no significant toxic effects on the heart, liver, spleen, lungs, or kidneys at a daily dose of 40 mg kg^−1^ (Figure S12, Supporting Information). Even when JAKi was administered four weeks post‐surgery, there was still a notable attenuation of OA progression (Figure S13, Supporting Information). In conclusion, tofacitinib, a clinically available JAKi, holds potential as a therapeutic agent for the treatment of OA.

### 2.6 JAKi Block IL‐6‐Induced SIRT1 Downregulation and Cell Senescence in Chondrocytes

**Figure 6:**
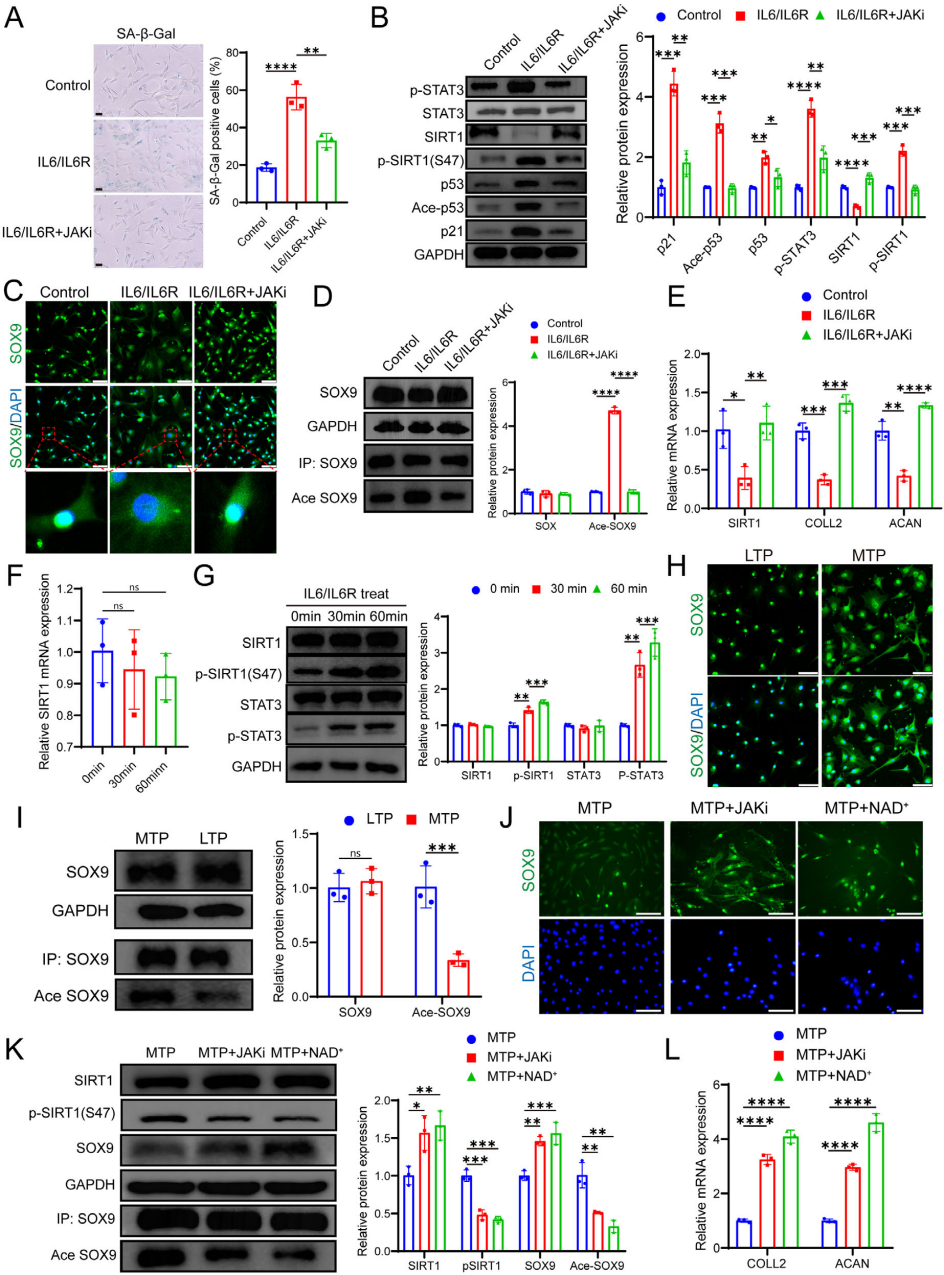
Treatment with IL‐6/IL‐6R complex induces chondrocyte senescence by inhibiting SIRT1. A) Chondrocytes were treated with Veh or IL‐6/IL‐6R complex, or IL‐6/IL‐6R complex + JAKi (3 µm) for 48 h. IL‐6/IL‐6R complex consisted of IL‐6 (100 ng mL^−1^) and IL‐6R (100 ng mL^−1^). SA‐β‐Gal staining was performed to detect cell senescence, followed by analysis of the percentage of SA‐β‐Gal‐positive cells. B) Western blotting was used to detect and quantify the protein levels of STAT3, p‐STAT3, SIRT1, p‐SIRT1(S47), p53, Ace‐p53, and p21 in chondrocytes. C) DAPI staining was used to visualize nuclei (blue fluorescence), while SOX9 expression was detected using an anti‐SOX9 antibody and Alexa Fluor 488‐conjugated secondary antibody (green fluorescence). The overlap of blue and green fluorescence was analyzed to assess alterations in the nuclear localization of SOX9. Scale bar: 100 µm. D) Following confirmation of SOX9 expression via immunoblotting of input samples, lysates were subjected to immunoprecipitation using an antibody specific to SOX9. The immunoprecipitated complexes were then analyzed by immunoblotting to detect SOX9 and acetylated lysine (AcetylK) residues. E) Chondrocytes were treated with Veh, IL‐6/IL‐6R complex, or IL‐6/IL‐6R complex plus JAKi (3 µm) for 48 h. RT‐qPCR was employed to quantitatively assess the expression levels of SIRT1, COL2A1, and ACAN mRNA. F) Chondrocytes were treated with Veh, IL‐6/IL‐6R complex for 30 min, or IL‐6/IL‐6R complex for 60 min. RT‐qPCR was employed to quantitatively assess the expression levels of SIRT1 mRNA. G) Western blotting was utilized to detect and quantify the protein expression levels of SIRT1, p‐SIRT1(S47), p‐STAT3, and total STAT3 in chondrocytes. H) Primary cartilage cells from LTP or MTP cartilage were measured for SOX9 expression, and the nucleus was stained with DAPI. Scale bar: 100 µm. I) Western blotting was used to detect and quantify SOX9 protein levels in LTP or MTP cartilage. The immunoprecipitated complexes were analyzed for SOX9 and acetylated lysine (AcetylK) residues. J) The MTP‐derived chondrocytes were treated with Veh, JAKi (3 µm), and NAD^+^(10 mm), respectively, for 48 h. The expression of SOX9 was detected by immunofluorescence. Scale bar: 100 µm. K) Western blotting was utilized to detect and quantify the protein expression levels of SIRT1, p‐SIRT1(S47), and total SOX9 in chondrocytes. The immunoprecipitated complexes were then analyzed by immunoblotting to assess the presence of SOX9 as well as acetylated lysine (AcetylK) residues. L) RT‐qPCR was employed to assess the expression levels of COLL2 and ACAN mRNA quantitatively. Quantitative data are presented as mean ± S.D. Statistical analysis was performed using one‐way ANOVA with Tukey's multiple comparisons (A, F) and two‐way ANOVA with Sidak's multiple comparisons. *p < 0.05, **p < 0.01, ***p < 0.001, ****p < 0.0001, ns, no significant.

Although IL‐6 is known to induce cellular senescence and is commonly used as a model for screening anti‐aging drugs,^[^ ^15^ ^]^ the specific mechanism by which IL‐6 pathway activation leads to senescence remains unclear. In this study, we treated human chondrocytes with the IL‐6/sIL‐6R complex and observed that it induces cellular senescence, likely through the downregulation of SIRT1 and the activation of the p53/p21 signaling pathway (**Figure** **6**A,B). This treatment also resulted in the downregulation of SIRT1, which increased SOX9 acetylation, hindered its nuclear localization, and subsequently reduced collagen synthesis (Figure 6C–E). Notably, JAKi treatment was able to block the IL‐6 pathway‐induced cellular senescence and the associated decline in cartilage collagen synthesis (Figure 6B–E).

Interestingly, early treatment with the IL‐6/sIL‐6R complex did not significantly reduce the mRNA and protein levels of SIRT1 but did increase the phosphorylation level of SIRT1 at the S47 site (Figure 6F,G). Previous studies have shown that phosphorylation of SIRT1 at the S47 site reduces its deacetylase function,^[^ ^21^ ^]^ but this has not been explored in the context of OA. In 293T cell lines, overexpression of human SIRT1 (hSIRT1), the S47A mutant (hSIRT1‐S47A), and the S47D mutant (hSIRT1‐S47D) revealed that hSIRT1‐S47A exhibited enhanced deacetylase activity and protein stability, whereas hSIRT1‐S47D was more prone to degradation (Figure S14, Supporting Information). Furthermore, to confirm the direct binding of SIRT1 with SOX9 under stable conditions, we performed co‐immunoprecipitation (CO‐IP) experiments to confirmed that SIRT1 interacts with SOX9, and SIRT1 modifies SOX9 through deacetylation (Figure S15, Supporting Information).

In human OA cartilage samples, we validated that SIRT1 expression was reduced in degenerated cartilage, while pSIRT1(S47) expression was increased (Figure S16, Supporting Information). Although SOX9 protein levels did not differ significantly between degenerated and relatively healthy cartilage, chondrocytes from degenerated cartilage displayed impaired SOX9 nuclear localization, associated with increased SOX9 acetylation (Figure 6H,I). JAKi treatment promoted SIRT1 stability, thereby reducing SOX9 acetylation and facilitating its nuclear localization (Figure 6J–L), uncovering a novel mechanism by which JAKi mitigates OA.

### 2.7 JAKi Can Induce Apoptosis of p21‐Positive Senescent Cells in OA Degenerative Cartilage

**Figure 7:**
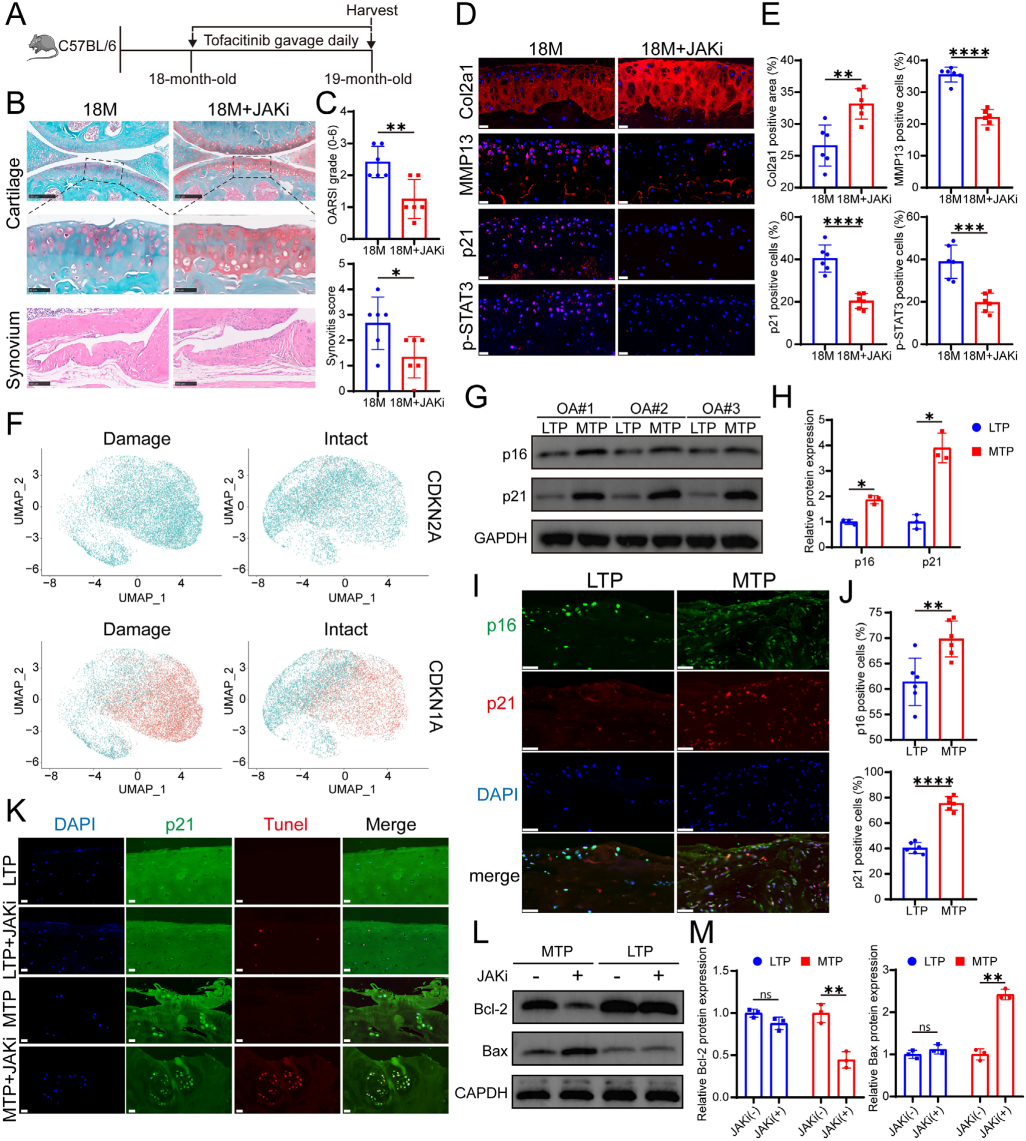
JAKi treatment reduced p21‐positive cells in the knee joint of aged mice and induced apoptosis of p21‐positive cells in human OA cartilage blocks. A) Schema illustrating the experimental setup for assessing the effect of JAKi on cartilage degeneration in aged mice (for B–E). Eighteen‐month‐old C57BL/6 mice were treated with either vehicle (Veh) or JAKi (tofacitinib) via gavage daily for four weeks. B) Knee cartilage from 19‐month‐old mice was stained with SO&FG and synovial tissue with H&E. (*n* = 6 mice per group). Scale bar: top: 250 µm, middle: 50 µm, bottom: 100 µm. C) Quantification of OARSI and synovitis scores. D) Immunofluorescence staining of Col2a1, MMP13, p21, and p‐STAT3 in mice knee cartilage, with DAPI‐stained nuclei. Scale bar: 20 µm. E) Analysis of Col2a1‐positive area and the proportion of MMP13, p21, and p‐STAT3‐positive cells. F) Dot plots showing the expression of p21 (encoded by CDKN1A) and p16 (encoded by CDKN2A) in damaged and intact cartilage samples on the UMAP map. Red indicates cells with high expression of p21 or p16, while blue represents cells with low expression of these markers. The dataset originates from Lv Z, Han J, Li J, et al.’s study titled “Single cell RNA‐seq analysis identifies ferroptotic chondrocyte cluster and reveals TRPV1 as an anti‐ferroptotic target in osteoarthritis,” published in EBioMedicine. The single‐cell sequencing experiment was designed by using the medial and lateral condylar cartilage of the femur in 3 patients with OA. The single‐cell sequencing information was acquired and utilized with the permission of the research team involved. G,H) Western blotting detected and quantified the protein levels of p21 and p16 in MTP and LTP cartilage from 3 OA patients (*n* = 3). I) The expression of p16 and p21 in chondrocytes was evaluated by immunofluorescence staining in LTP or MTP cartilage (*n* = 6). Scale bar: 50 µm. J) Immunofluorescence staining analyzed the proportion of p16 and p21 positive cells (*n* = 6). K) LTP and MTP cartilage blocks from the same TKA patient's tibial plateau were aseptically dissected into 4 mm × 4 mm pieces and cultured for 6 days in media with or without JAKi. Double immunofluorescence staining for Tunel and p21 with DAPI nuclear counterstaining. Scale bar: 50 µm. L,M) Western blotting detected and quantified the protein levels of Bcl‐2 and Bax in LTP and MTP cartilage blocks with or without JAKi treatment. Quantitative data are presented as mean ± S.D. Statistical analysis was performed using a two‐sided unpaired Student's t‐test in (C) and (E), and a two‐sided paired Student's t‐test in (H), (J), and (M). *p < 0.05, **p < 0.01, ***p < 0.001, ****p < 0.0001, ns, no significant.

To further evaluate the therapeutic potential of JAKi in treating degenerated cartilage, we administered JAKi to aged mice (**Figure** **7**A). The results showed that aged mice exhibited moderate cartilage degeneration, and JAKi treatment notably increased cartilage matrix content in the knee joint cartilage (Figure 7B–E). While a significant number of p21‐positive senescent cells were present in the knee joint cartilage of aged mice, their numbers significantly decreased after one month of JAKi treatment (Figure 7D,E). This led us to investigate the mechanism by which JAKi reduces senescent cells in vivo. Considering the role of the JAK/STAT3/BCL2 pathway as an anti‐apoptotic mechanism, we discovered that JAKi treatment induced apoptosis in primary cells derived from human OA‐degenerated cartilage, whereas cells from relatively healthy cartilage showed minimal apoptosis (Figure S17, Supporting Information). To determine the suitability of using p21 or p16 as markers for identifying senescent cells within degenerative cartilage, we further compared degenerative and relatively healthy cartilage using self‐controlled methods (Figure 7F–J). Single‐cell sequencing revealed a higher proportion of p21‐positive cells in degenerated cartilage, with a relatively small difference in p16‐positive cells compared to the control sample (Figure 7F). Western blot analysis revealed significant differences in the expression levels of both p21 and p16 between the two groups, with the expression difference of p21 (expressed in log scale, 2.901 [1.933–3.869]) being notably greater than that of p16 (expressed in log scale, 0.8702 [0.2761–1.464]) (Figure 7G,H). Subsequent immunofluorescence staining corroborated these findings, showing a significantly more significant difference in the proportion of p21‐positive cells (expressed in log scale, 35.06% [27.94–42.18%]) than in p16‐positive cells (expressed in log scale, 8.417% [4.227–12.61%]) between the two groups (Figure 7I,J). In particular, the degenerative group exhibited a higher proportion of cells that were double‐positive for both p21 and p16 compared to the relatively healthy cartilage. Consequently, we selected p21 as the primary marker for senescent cells. Further experiments revealed that JAKi treatment induced apoptosis in p21‐positive cells within degenerated cartilage blocks (Figure 7K), likely by disrupting the BCL2/Bax balance (Figure 7L,M).

### 2.8 Targeted Elimination of p21‐Positive Senescent Cells Alleviates OA Progression in Mice

**Figure 8:**
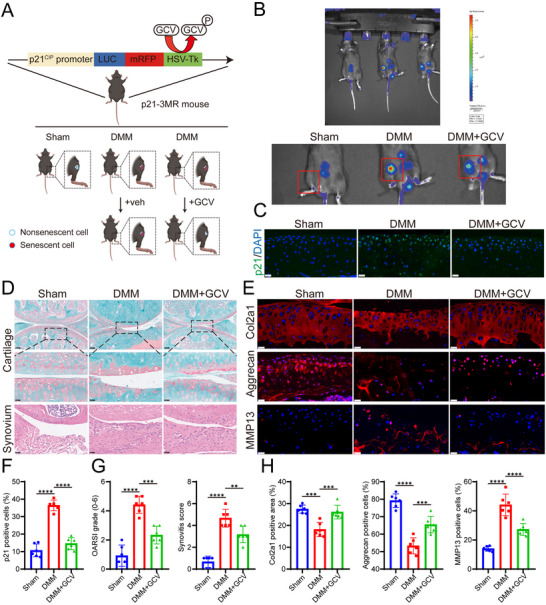
Clearance of p21‐positive senescent cells alleviates OA progression in p21‐3MR mice. A) Schema illustrating the experimental setup for clearing p21‐positive senescent cells using ganciclovir (GCV) to mitigate OA induced by destabilization of the medial meniscus (DMM) surgery in p21‐3MR mice. Mice were gavaged with either vehicle (Veh) or GCV starting 7 days post‐surgery, with weekly 7‐day treatments followed by one‐week breaks, for a total of 6 cycles. B) Luminescence images of mice (sham, DMM + Veh, DMM + GCV) at 42 days post‐surgery. C) Immunofluorescence staining of knee cartilage for p21 to evaluate the clearance of p21‐positive cells. Scale bar: 20 µm. D) Cartilage damage and synovitis were evaluated at 6 weeks using SO&FG and H&E staining. Scale bar: top: 100 µm, middle: 25 µm, bottom: 20 µm. E) Immunofluorescence staining for Col2a1, aggrecan, and MMP13, with DAPI‐stained nuclei in knee cartilage (*n* = 6 mice/group). Scale bar: 20 µm. F) Quantification of the proportion of p21‐positive cells by immunofluorescence staining (*n* = 6). G) Quantification of OARSI and synovitis scores. H) Analysis of Col2a1‐positive area and the proportion of aggrecan and MMP13‐positive cells in mice knee cartilage. Abbreviations: GCV, ganciclovir; Veh, vehicle. Statistical analysis was performed using one‐way ANOVA with Tukey's multiple comparisons. *p < 0.05, **p < 0.01, ***p < 0.001, ****p < 0.0001, ns, no significant.

As consistent with our findings, recent studies have suggested that p21 is a more specific marker for senescent cells than p16,^[^ ^22^ ^]^ making it a potentially more precise target for clearing senescent cells in OA. Although previous research has shown that eliminating p16‐positive senescent cells can alleviate OA,^[^ ^16a^ ^]^ no studies have yet investigated the potential of targeting p21‐positive senescent cells for OA treatment. In this study, we introduced a p21‐3MR mouse model and administered GCV injections to achieve targeted clearance of p21‐positive senescent cells (**Figure** **8**A). In vivo imaging system (Spectrum IVIS) results revealed a significant increase in fluorescence intensity in the knee joints of mice following DMM surgery, indicating an accumulation of senescent cells. GCV treatment, however, reduced this fluorescence intensity, suggesting a decrease in the number of senescent cells (Figure 8B). Histological staining showed that the OA group exhibited obvious cartilage degeneration and a high number of p21‐positive cells in the knee cartilage. In contrast, GCV treatment significantly mitigated cartilage degeneration and reduced the presence of p21‐positive senescent cells (Figure 8C–H). In conclusion, these findings suggest that the targeted clearance of p21‐positive senescent cells can effectively alleviate the progression of OA.

## 3 Discussion

OA is a whole‐joint disease affecting multiple tissues, but the progressive loss of articular cartilage is a key feature.^[^ ^5^, ^23^ ^]^ Its etiological factors include aging, joint overuse or injury, obesity, and heredity.^[^ ^5c^ ^]^ The molecular mechanisms of OA are still unclear. There is currently no cure for OA.^[^ ^23^, ^24^ ^]^ Clinical treatments have long focused on relieving joint pain rather than halting disease progression. Recently, strategies have shifted to early prevention and delaying OA progression before major damage.^[^ ^5c^ ^]^ Low‐grade chronic inflammation in the joint can promote OA progression, such as TNF‐α, IL‐1, IL‐6, and IL‐8, which were shown to trigger joint inflammation, which leads to cartilage degeneration.^[^ ^5^, ^8^ ^]^ Traditional anti‐inflammatory drugs, such as COX inhibitors (e.g., celecoxib), are commonly used to alleviate pain and inflammation in OA patients. However, their long‐term use does not impede joint remodeling or disease progression and may lead to adverse effects, including gastrointestinal issues, kidney damage, and cardiovascular risks. Therefore, finding alternative methods to effectively inhibit joint inflammation and prevent cartilage degeneration without these side effects is an important direction for mitigating the progression of OA.^[^ ^6a^ ^]^

Chronic low‐grade inflammation, driven by factors such as abnormal mechanical stress,^[^ ^25^ ^]^ age‐related changes,^[^ ^26^ ^]^ and metabolic conditions like obesity and metabolic syndrome,^[^ ^27^ ^]^ significantly contributes to OA pathogenesis. Previous studies have demonstrated that this inflammation primarily leads to cartilage matrix degradation^[^ ^5b^ ^]^ and cellular senescence,^[^ ^25^ ^]^ with inflammatory factors stimulating the production of matrix‐degrading enzymes such as MMPs. However, the elevated levels of inflammatory factors in joint fluid do not fully explain the more severe degeneration of the medial tibial plateau cartilage in OA patients. To address this clinical challenge, we investigated the activation of the IL‐6 pathway in cartilage with varying degrees of degeneration. The IL‐6/JAK2 signaling pathway plays a pivotal role in the inflammatory and catabolic processes underlying OA pathogenesis.^[^ ^11^, ^12^ ^]^ Upon binding of IL‐6 to its receptor (IL‐6R),^[^ ^19^ ^]^ downstream activation of the JAK2/STAT3 cascade leads to the transcription of pro‐inflammatory cytokines and matrix‐degrading enzymes, such as MMPs and a disintegrin and metalloproteinase with thrombospondin motifs (ADAMTS).^[^ ^12c^ ^]^ This pathway has been implicated in promoting synovial inflammation, cartilage degradation, and subchondral bone remodeling, which are hallmarks of OA progression.^[^ ^12c^ ^]^ Our results revealed abnormally high expression of IL‐6R, p‐JAK2, and p‐STAT3 in severely degraded cartilage. By knocking out IL‐6R or JAK2, we were able to alleviate OA progression due to aging and joint instability in murine models. Further validation came from studying gp130 point mutant mice,^[^ ^28^ ^]^ which exhibit accelerated OA progression due to increased STAT3 pathway activation. This study confirms the pathogenic role of aberrant IL‐6R/p‐JAK2 activation in knee OA and underscores the potential of targeting the IL‐6R/JAK2 pathway as a therapeutic approach for OA.

Previous studies have shown that knocking out the IL‐6 gene or administering IL‐6 antibodies intra‐articularly could significantly alleviate OA progression.^[^ ^10^, ^29^ ^]^ Although anti‐IL‐6R antibodies, such as tocilizumab, have demonstrated promising anti‐inflammatory effects and symptom relief in certain OA patients,^[^ ^1^, ^2^ ^]^ the therapeutic response varies across individuals, with some patients experiencing limited benefits. IL‐6 is currently believed to contribute to cartilage catabolism, primarily by enhancing the synthesis of MMPs.^[^ ^12b^ ^]^ While IL‐6 has been documented to promote cellular senescence, the underlying mechanism has remained unclear.^[^ ^15^ ^]^ Our findings indicate that IL‐6 drives the activation of the p53/p21 pathway by downregulating SIRT1, thereby inducing cellular senescence. Additionally, the IL‐6/sIL‐6R complex treatment leads to decreased SIRT1 levels, which increases the acetylation of SOX9. This modification hampers SOX9's nuclear entry, negatively impacting cartilage matrix synthesis.^[^ ^30^ ^]^ We also found that IL‐6/IL‐6R complex treatment promotes SIRT1 phosphorylation at the S47 site, leading to its degradation. Inhibition of this phosphorylation enhances SIRT1's deacetylase activity and slows its degradation.^[^ ^21^, ^31^ ^]^ Interestingly, our study observed abnormally low SIRT1 expression and high pSIRT1(S47) levels in degenerated OA cartilage. JAKi can block IL‐6‐induced senescence in chondrocytes and mitigate the reduction in collagen synthesis caused by SOX9's impaired nuclear translocation. Notably, in human OA cartilage, we made an intriguing discovery that chondrocytes from degenerated cartilage exhibit impaired nuclear translocation of SOX9, a defect mediated by increased acetylation of SOX9. Treatment with both JAKi and SIRT1 agonists promotes the re‐entry of SOX9 into the nucleus. In conclusion, compared to current anti‐inflammatory drugs, such as COX inhibitors (e.g., celecoxib), JAK inhibitors demonstrate a dual role in regulating both cartilage synthesis and degradation,^[^ ^32^ ^]^ offering a promising therapeutic approach for OA treatment.

While JAKi suppresses the SASP in senescent cells,^[^ ^17a^ ^]^ current research mainly focuses on its inhibitory effect on the inflammatory response, like down‐regulating IL‐6 secretion^[^ ^32^ ^]^ and reducing MMP production for collagen degradation to prevent OA progression.^[^ ^33^ ^]^ However, there's no literature on its induction of apoptosis in senescent chondrocytes.^[^ ^17b^ ^]^ In our study, treating elderly mouse knee cartilage with JAKi significantly reduced the number of senescent chondrocytes. However, the disappearance of a large number of senescent chondrocytes cannot be fully explained by SASP inhibition alone.^[^ ^17a^ ^]^ Given that the JAK/STAT3/BCL2 pathway has been identified as anti‐apoptotic, we hypothesized that JAKi might induce apoptosis in senescent cells in vivo. To test this hypothesis, we then treated human OA‐degraded cartilage with JAKi, which induced apoptosis in p21‐positive cells within the degraded tissue. This apoptosis may be linked to the disruption of the BCL2/Bax balance.^[^ ^31^ ^]^ Further analysis using single‐cell sequencing and immunofluorescence staining of human tissue samples confirmed the presence of a significant number of p21‐positive senescent cells in degenerated cartilage. Given that p16‐positive cells are more abundant in relatively healthy cartilage, using p21 as a marker for senescent cells in degenerated cartilage provides greater specificity. Although the impact of targeting p21‐positive senescent cells in OA has not been previously reported, studies have shown that selectively eliminating p21‐positive cells offers better specificity in clearing senescent cells and has demonstrated efficacy in alleviating osteoporosis.^[^ ^22^, ^35^ ^]^ In our experiment, we successfully generated p21‐3MR mice to selectively eliminate p21‐positive senescent cells, which resulted in a mitigated progression of OA. These findings highlight the potential of targeting p21‐positive senescent cells as a promising therapeutic approach for OA.

In conclusion, this study highlights the significance of the IL‐6R/JAK2 pathway in the activation of the IL‐6 signaling cascade within degenerative cartilage in OA. Targeting IL‐6R/JAK2 has the potential to mitigate the progression of OA. Notably, the JAKi was found to promote SIRT1 protein stability in OA chondrocytes, thereby inhibiting the acetylation of SOX9 and facilitating its nuclear translocation, which in turn boosts collagen synthesis. Additionally, JAKi induced apoptosis in p21‐positive senescent cells, and their targeted elimination significantly alleviated OA progression. These findings position JAKi as a promising therapeutic candidate for OA treatment.

## 4 Experimental Section

### Human Samples

Human knee OA cartilage samples were sourced from 25 patients (15 females and 10 males, aged 55–70 years) who underwent total knee arthroplasty (TKA) surgeries for clinically and radiologically diagnosed OA. Patients with a history of rheumatic arthropathies or knee infections were carefully excluded from the study. The major load‐bearing areas of the lateral tibial plateau (LTP) were classified as relatively healthy cartilage, while those of the medial tibial plateau (MTP) were considered degenerative. Detailed patient information is provided in Table S1 (Supporting Information). The study was conducted with the approval of the Ethics Committee at Henan Provincial People's Hospital (MR‐41‐22‐011500).

### Animal

Animal experiments followed the regulations and guidelines of the Animal Care and Use Committee of Heymouse Biological Technology Co., Ltd (2022050501). gp130 (Y757F/Y757F) mice were obtained from Shanghai Model Organisms, while IL6R knockout (IL6R^−/−^) mice, IL6 knockout (IL6^−/−^) mice, JAK2^flox/flox^ mice, CAG‐Cre‐ERTM mice, C57BL/6 wild‐type (WT) mice, p21‐3MR mice, and SD rats were sourced from Cyagen Biosciences Inc. To generate CAG‐CreERTM; JAK2^flox/flox^ mice (JAK2 KO), JAK2^flox/flox^ mice were mated with CAG‐CreERTM mice to produce CAG‐CreERTM; JAK2^flox/+^ mice, which were subsequently mated with JAK2^flox/flox^ mice. Littermate JAK2^flox/flox^ mice (in the absence of CAG‐CreERTM) served as JAK2 KO mice control. Tamoxifen was administered prior to model induction to ensure the proper activation of Cre recombinase and efficient gene deletion in the KO mice. At 9 weeks or 18 months of age, mice received daily intraperitoneal injections of tamoxifen (40 mg kg^−1^ diluted to 10 mg mL^−1^ in corn oil) for 5 days to activate Cre recombinase activity of CAG‐CreERTM and induce JAK2 deficiency. For tamoxifen control, JAK2‐intact mice (without CAG‐CreERTM) also received tamoxifen injections. Genotyping was performed via PCR using tail DNA. Previous literature demonstrated that treatment with tamoxifen alone or the CAG‐CreERT2 allele alone has no effect on OA outcomes in the DMM model. All mice used in the experiments were males on the C57BL/6 background strain, and littermate mice were randomly assigned to groups. For OA experiments, 10‐week‐old male transgenic mice, C57BL/6 mice, and 12‐week‐old SD rats were utilized. For aged‐OA studies, 3‐month‐old and 18‐month‐old male transgenic mice were employed. Sample sizes for each experiment were determined based on previous experience, and no mice were excluded from the analysis.

All mice and rats were housed under pathogen‐free conditions with a maximum of five animals per cage. They had free access to water and food and were maintained at a constant temperature of 23–25 °C, with circulating air, 45–65% humidity, and a 12‐h light/dark cycle. Prior studies showed increased OA severity in male mice compared to females following DMM surgery. Therefore, male mice or rats were used in all experiments and were randomly assigned to either control or treatment groups for OA evaluation. In determining the sample size of animal experiments, discrepancies were noted in the sizes utilized across various studies in the realm of OA, ranging from *n* = 5 to *n* = 11. The choice of *n* = 6 per group was informed by the dual objectives of securing statistically significant results and adhering to ethical standards.

Ten‐week‐old male C57BL/6 mice, gp130 (Y757F/Y757F), IL6^−/−^ mice, IL6R^−/−^ mice, JAK KO mice, and littermate control mice were subjected to surgical DMM surgery to induce OA. Briefly, the mice were anesthetized and underwent aseptic surgery on their right knees. The joint capsule medial to the patellar tendon was opened, the intercondylar region was exposed, and the medial meniscotibial ligament was cut. The joint capsule and skin were then closed. For the 12‐week‐old male S.D. rats, the DMM operation was combined with ACLT, meaning the anterior cruciate ligament was severed concurrently with the DMM procedure. In sham‐treated animals, the joint capsule was opened but the ligament was left intact. Mice and S.D. rats were euthanized with carbon dioxide or isoflurane (1.2–2% in O2) for knee‐joint specimen collection at 4 or 8 weeks post‐surgery.

To assess the effect of recombinant IL‐6R protein on cartilage degeneration, an insulin syringe needle was sagittally inserted into the intercondylar area of the rat's knee. A 50 µL dose of recombinant IL‐6R protein (100 ng mL^−1^) was injected weekly for 4 weeks following ACLT + DMM surgery or sham operation, with normal saline used as the control.

Three OA animal models were utilized to investigate the effect of a JAK inhibitor (Tofacitinib, JAKi) on alleviating OA. For the prevention model of surgery‐induced OA, the OA rats were orally administered with 10 mg kg^−1^ tofacitinib or an equal amount of DMSO 3 days after ACLT + DMM surgery. For the treatment model of surgery‐induced OA, the OA rats were orally administered with 10 mg kg^−1^ tofacitinib or an equal amount of DMSO 4 weeks after ACLT + DMM surgery. For age‐induced OA, 18‐month‐old mice were orally administered 10mg kg^−1^ of tofacitinib or an equal amount of DMSO for 1 month.

### ^18^F‐FDG PET Scan Analysis

An ^18^F‐FDG PET scan was performed on SD rats using a MicroPET/CT scanner (Inviscan, France) 8 weeks after ACLT + DMM surgery. After an overnight fast, rats received an intravenous injection of 11.1 MBq ^18^F‐FDG in 300.0 µL H_2_O via the tail vein, followed by a 1‐h uptake interval. Anesthetized with 2.0% isoflurane, rats underwent a 10‐min static PET/CT scan. Image analysis was conducted using Inveon Research Workplace software (Siemens, USA), calculating SUVmean and SUVmax in ROIs. Joint SUVmax was adopted in this study.

### Statistical Analysis

All statistical analyses were performed using Prism GraphPad 8.0 and R software (version 4.3). Results are presented as mean ± standard deviation (S.D.), as detailed in the figure legends. Differences between two groups were assessed using a two‐sided unpaired Student's t‐test or a two‐sided paired Student's t‐test, as appropriate. For comparisons involving more than two groups, one‐way ANOVA with Tukey's multiple comparison tests was employed. To evaluate differences among multiple groups and indicators, two‐way ANOVA followed by Sidak's multiple comparisons tests was used. Statistical significance was determined with a threshold of *P* < 0.05 (*p < 0.05, **p < 0.01, ***p < 0.001, ****p < 0.0001), and “ns” denotes no significant difference.

## Conflict of Interest

The authors declare no conflict of interest.

## Author Contributions

X.Z., J.L., F.L., and Y.Z. contributed equally to this work. Conceptualization, and design of the study was performed by X.Z., J.L., F.L., and Y.G.; Data curation by X.Z., J.L., and J.Y.; Methodology was performed by J.Y., Y.Z., and B.S.; Software was developed by J.L., J.Y., and Z.W.; Funding acquisition was led by X.Z., H.S., and Y.G. X.Z., J.M.L., J.X.Y., and Y.Z.G. designed the experiments and directed the study. X.Z., F.L., Y.Z., J.M.L., Z.Q.W., S.X., C.H.M., and B.S. conducted experiments, data analysis, and interpretation. X.Z. and J.M.L. wrote the manuscript. All authors have read and verified the underlying data and approved the final version of the manuscript.

## Supporting information



Supporting Information

## Data Availability

The data that support the findings of this study are available in the supplementary material of this article.
