# Supplementary material for: Targeting p21‐Positive Senescent Chondrocytes via IL‐6R/JAK2 Inhibition to Alleviate Osteoarthritis
Source: Adv Sci (Weinh). 2025 Jan 23;12(11):2410795. doi: 10.1002/advs.202410795 (PMC11923994; doi:10.1002/advs.202410795)
Supplement: Supplementary file 1 — Supporting Information [file ADVS-12-2410795-s001.docx]

**Supplementary Materials for**

**Targeting p21-Positive Senescent Chondrocytes via IL-6R/JAK2 Inhibition to Alleviate Osteoarthritis**

Authors: Xiang Zhao*^1^, Jieming Lin*^2^, Feng Liu*^3^, Yu Zhang*^1^, Bo Shi^4^, Chunhui Ma^5^, Ziqi Wang^6^, Song Xue^7^, Qingrong Xu^2^, Hongda Shao^&,8^, Jingxing Yang^&,9^, Yanzheng Gao^&,1^

Correspondence to: shd158101305@126.com; tom123yang@sjtu.edu.cn; yanzhenggaohn@163.com.

**The file includes:**

Materials and Methods,

Supplementary Table S1 and Table S2,

Supplementary Figure S1-S18 with their legends.

**Materials and Methods**

**Senescence-associated β-galactosidase (SA-β-Gal) staining**

Cytochemical staining for SA-β-Gal was performed using a commercially available SA-β-Gal staining kit (Beyotime, Shanghai, China, C0602) to assess senescence in chondrocytes by evaluating β-galactosidase activity. For adherent chondrocytes or cartilage tissue frozen section, samples were fixed with 0.2% glutaraldehyde for 15 min at room temperature following the manufacturer's instructions. X-gal staining solution of pH6.0 was then applied to the sample and incubated at 37℃ overnight. The percentage of SA-β-Gal positive cells in four randomly selected fields per treatment was quantified (n=6).

**RNA isolation, cDNA synthesis, and RT-qPCR**

Total RNA was extracted from primary articular chondrocytes (1×10^6^) and cartilage samples (0.05 g) using Trizol reagent (Biosharp, BS258A) and reverse transcribed into cDNA with a PrimeScript™ RT Reagent Kit (Takara). RT-qPCR was performed using a real-time qPCR system (ABI, Life Technologies) with SYBR Green Master Mix (YEASEN, 11202ES03) to measure the expression of IL-6R, SIRT1, Col2a1, and aggrecan. Samples were run in triplicate and normalized to GAPDH using the 2^-ΔΔCT^ method. Primer sequences are detailed in Supplementary Table S2.

**Culture of primary articular chondrocytes and cartilage explants**

Primary articular chondrocytes were obtained by digesting articular cartilage tissue from knee OA patients' LTP or LTP cartilage with 0.2% type 2 collagenase (Sigma, USA). The detailed procedure was processed as previously described. For the follow-up experiments, we used first-passage chondrocytes from knee OA patients' cartilage. Chondrocytes were treated with IL-6/sIL-6R complex, or IL-6/IL-6R complex +JAKi (3 μM) for 48 hours. IL-6/sIL-6R complex consist of IL-6 100 ng/mL and IL-6R 100 ng/mL (R&D Systems). LTP and MTP cartilage blocks from the same TKA patient's tibial plateau were aseptically dissected into 4 mm×4 mm pieces and cultured for 6 days in a cell culture medium with or without JAKi (3 μM). The medium for the cartilage blocks was changed daily.

**Construction of SIRT1 mutants**

The cDNA sequence of Homo sapiens sirtuin 1 (SIRT1), transcript variant 1 (NM_012238.5), was obtained from NCBI. After deleting the stop codon, a Flag tag sequence (GATTACAAGGACGACGATGACAAG) was fused to the C-terminus. Mutations were introduced at the S47 site, where AGC was changed to GCC to generate the S47A variant and to GAC to generate the S47D variant. For SOX9, the transcript (NM_000346.4) was obtained from NCBI. After deleting the stop codon, a Myc tag sequence (GAACAAAAACTCATCTCAGAAGAGGATCTG) was fused to the C-terminus. The designed sequences were then submitted to GENEWIZ (Suzhou, China) and cloned into the pcDNA3.1 vector. Transient transfections of 293T cells were performed using Lipofectamine 2000 (Cat: 11668027, Thermo Fisher Scientific), with pcDNA3.1 or three other vectors used for overexpressing hSIRT1, S47A, and S47D.

**Magnetic Resonance Imaging**

Both limbs were harvested at sacrifice and immediately stored at 4°C prior to the acquisition of magnetic resonance (MR) images within 12 hours of sacrifice. Scans were performed using a 3T T_2_-weighted sequence. The parameters were set as follows: TR=3000 ms, TE=100 ms, and slice thickness=1 mm.

**Micro-Computed Tomography (micro-CT) analysis**

The knee joint samples from SD rats were first fixed in 4% PFA for 24 hours and then scanned using high-resolution micro-CT (mCT80; Shanghai Huaiyu Biotechnology Co., LTD) with a voxel size of 6 μm, a voltage of 70 kV, an intensity of 142 μA, and an exposure time of 1000 ms. The scans comprised approximately 1300 slices spanning the femur, tibia, and knee joint. The region of interest was defined to include the entire tibial subchondral bone compartment. Three-dimensional structural parameters, including total tissue volume (trabecular and cortical bone) and trabecular thickness, were analyzed using manufacturer-specific analysis tools.

**Western blotting (WB) analysis**

The total proteins from both human cartilage samples and human primary chondrocytes were extracted utilizing a radioimmunoprecipitation assay (RIPA) Lysis buffer (Beyotime, P0013B), supplemented with phenylmethanesulfonyl fluride (PMSF, 1mM) (Beyotime, ST507). The samples were then centrifuged at 12000 g for 15 min at 4℃. The supernatant was collected, and the protein concentration was determined using a BCA protein assessment kit (Beyotime, P0010). Equal quantities (20 μg) of protein from each sample were separated through sodium dodecyl sulfate-polyacrylamide gel electrophoresis (SDS-PAGE). They transferred to polyvinylidene fluoride (PVDF) membranes, which were blocked with 5% dry milk in TBST buffer and incubated with primary antibodies against IL-6 (affinity, DF6087), IL-6R (affinity, DF6466), gp130 (affinity, AF6291), p-JAK2 (CST, 3771), STAT3 (CST, 9139), p-STAT3 (CST, 9145), p16 (Abcam, ab108349), P21 (Abcam, ab220206 and ab107099), p53 (affinity, AF0879), Ace p53 (affinity, AF3744), Bcl2 (Affinity, AF6139), Bax (CST, 2772), SIRT1(Abcam, ab110304 and Affinity, DF6033), pSIRT1(Affinity, AF3474), and SOX9 (Abcam, ab185966 and SAB, 49070) overnight at 4℃. After washing, the membranes were incubated with horseradish peroxidase-conjugated secondary antibodies for 1 h at room temperature, and the membranes were treated with an enhanced luminol-based detection reagent (Santa Cruz, USA) before exposure. The gray value of GAPDH was used to normalize total protein. The relative protein expression was measured using the Image J software.

**Histological analysis**

Tissue samples were fixed in 4% paraformaldehyde buffered with phosphate-buffered saline (pH 7.4) for 24 h at 4 °C. Specimens were decalcified with 10% EDTA (pH 7.4) for 3 weeks at 37°C, embedded in paraffin and 4-µm thick sagittal sections were cut. H&E and Safranin O/Fast Green staining were performed according to standard protocols. Safranin O/Fast Green staining slides were used to evaluate cartilage degeneration by the Osteoarthritis Research Society International (OARSI) scoring system. H&E slides were used to evaluate synovial activation by Krenn’s synovitis scoring system. Each section was assessed by three blinded, independent graders, and the mean score was used for statistical analysis.

**Immunofluorescence (IF) analysis**

Paraffin-embedded knee articular tissue sections from patients, rats, or mice were deparaffinized, hydrated, permeabilized, and subjected to antigen retrieval. Briefly, the tissue sections were incubated in xylene for approximately 15 minutes to remove the paraffin wax, with two changes of xylene to ensure complete removal. Following deparaffinization, the sections were gradually rehydrated by passing them through a graded ethanol series (100%, 95%, 70%, and 50%) for 5 minutes each. Subsequently, the tissue sections were washed twice with distilled water for 5 minutes each. Antigen retrieval was performed to unmask antigens potentially masked during fixation. The tissue sections were heated in citrate buffer (pH=6.0) or EDTA buffer (pH=8.0) in a water bath at approximately 95°C for 20 minutes, followed by cooling to room temperature. After antigen retrieval, the slides were rinsed thoroughly with Tris-buffered saline to remove any residual retrieval solution. Then the knee articular tissue was blocked with 5% serum in PBS for 1 hour and then incubated overnight at 4°C with primary antibodies against: IL-6 (Affinity, DF6087), IL-6R (human, Abcam, ab271042; mouse, Affinity, DF6466; rat, Affinity, DF2530), gp130 (Affinity, AF6291), pJAK1 (CST, 74129), pJAK2 (CST, 3771), pJAK3 (CST, 5031), Col2a1 (Abcam, ab34712), MMP13 (Abcam, ab219620), p21 (Abcam, ab107099; eBioscience, 14-6715-63), p-STAT3 (CST, 9145), and pSIRT1 (S47) (Affinity, AF3474). Subsequently, the sections were incubated with secondary antibodies conjugated to Alexa Fluor 488 (Abcam, ab150077) or Alexa Fluor 594 (Abcam, ab150116) for 1 hour at room temperature in the dark. Nuclei were counterstained with DAPI, and images were captured using an Olympus BX63 microscope (Japan).

Quantification was performed by counting the number of positively stained cells in five fields of view from three consecutive cartilage sections in each group. The proportion of positive cells was assessed using ImageJ software. For cell immunofluorescence (IF) analysis, cells were fixed with 4% paraformaldehyde for 15 minutes at room temperature, followed by permeabilization with 0.1% Triton X-100 in PBS for 10 minutes. The cells were then incubated overnight at 4°C with primary antibodies against IL-6R (Abcam, ab271042) and SOX9 (Abcam, ab185966). Nuclei were stained with DAPI, and fluorescence images were acquired using a Leica DMi8 microscope (Germany).

**Marker gene analysis**

Single-cell RNA-seq data were requested and downloaded from the National Genomics Data Center (accession number: HRA002569). The data contained 17638 cells from 3 damaged cartilage samples and 3 corresponding intact cartilage samples from the same patients. Data were normalized and scaled using the SCTransform function from the Seurat package with default parameters. Gene markers in each group were identified with the "Find All Markers" function. "UMAP" was used to display the cell distribution.

**Supplementary Table S1**

**Table S1.** Characteristics of specimens from patients with OA.

| **NO.** | **Age** | **Gender** | **LTP**  **ICRS Grade** | **MTP**  **ICRS Grade** | **Joint** | **Use** |
| --- | --- | --- | --- | --- | --- | --- |
| 1 | 57 | Female | 1 | 3 | Knee (R) | IF/qPCR/WB |
| 2 | 67 | Male | 1 | 4 | Knee (L) | IF/qPCR/WB |
| 3 | 69 | Male | 1 | 3 | Knee (L) | IF/qPCR/WB |
| 4 | 67 | Female | 1 | 4 | Knee (R) | IF/qPCR/WB |
| 5 | 64 | Female | 1 | 4 | Knee (L) | IF/qPCR/WB |
| 6 | 58 | Female | 1 | 4 | Knee (R) | IF/qPCR/WB |
| 7 | 63 | Female | 1 | 3 | Knee (R) | IF/qPCR/WB |
| 8 | 63 | Male | 2 | 5 | Knee (L) | IF/qPCR/WB |
| 9 | 62 | Female | 1 | 3 | Knee (L) | IF/qPCR/WB |
| 10 | 62 | Female | 1 | 4 | Knee (L) | IF/qPCR/WB |
| 11 | 68 | Male | 1 | 3 | Knee (R) | IF/qPCR/WB |
| 12 | 67 | Female | 1 | 3 | Knee (L) | IF/qPCR/WB |
| 13 | 66 | Male | 1 | 3 | Knee (R) | IF/qPCR/WB |
| 14 | 58 | Female | 1 | 3 | Knee (L) | IF/qPCR/WB |
| 15 | 59 | Female | 1 | 3 | Knee (L) | IF/qPCR/WB |
| 16 | 56 | Female | 2 | 4 | Knee (R) | IF/qPCR/WB |
| 17 | 69 | Male | 1 | 3 | Knee (L) | IF/qPCR/WB |
| 18 | 61 | Female | 1 | 3 | Knee (R) | IF/qPCR/WB |
| 19 | 57 | Female | 1 | 4 | Knee (R) | IF/qPCR/WB |
| 20 | 65 | Male | 2 | 4 | Knee (R) | IF/qPCR/WB |
| 21 | 65 | Male | 2 | 4 | Knee (L) | IF/qPCR/WB |
| 22 | 59 | Male | 1 | 3 | Knee (L) | IF/qPCR/WB |
| 23 | 63 | Female | 1 | 3 | Knee (L) | IF/qPCR/WB |
| 24 | 68 | Male | 1 | 4 | Knee (R) | IF/qPCR/WB |
| 25 | 60 | Female | 1 | 3 | Knee (R) | IF/qPCR/WB |

ICRS: International Cartilage Repair Society; L: Left; R: Right; qPCR: quantitative; real-time PCR; WB: Western Blot; IF: immunofluorescence

**Supplementary Table S2**

**Table S2.** Primer sequences (5′-3′)

| Gene | Forward | Reverse |
| --- | --- | --- |
| GAPDH | ACCACAGTCCATGCCATCAC | TCCACCACCCTGTTGCTGTA |
| SIRT1 | TAGCCTTGTCAGATAAGGAAGGA | ACAGCTTCACAGTCAACTTTGT |
| IL-6R | CATGTGCGTCGCCAGTAGT | AGCTCAAACCGTAGTCTGTAGA |
| COL2A1 | TGGACGATCAGGCGAAACC | GCTGCGGATGCTCTCAATCT |
| ACAN | GTGCCTATCAGGACAAGGTCT | GATGCCTTTCACCACGACTTC |

**Supplementary Figures**


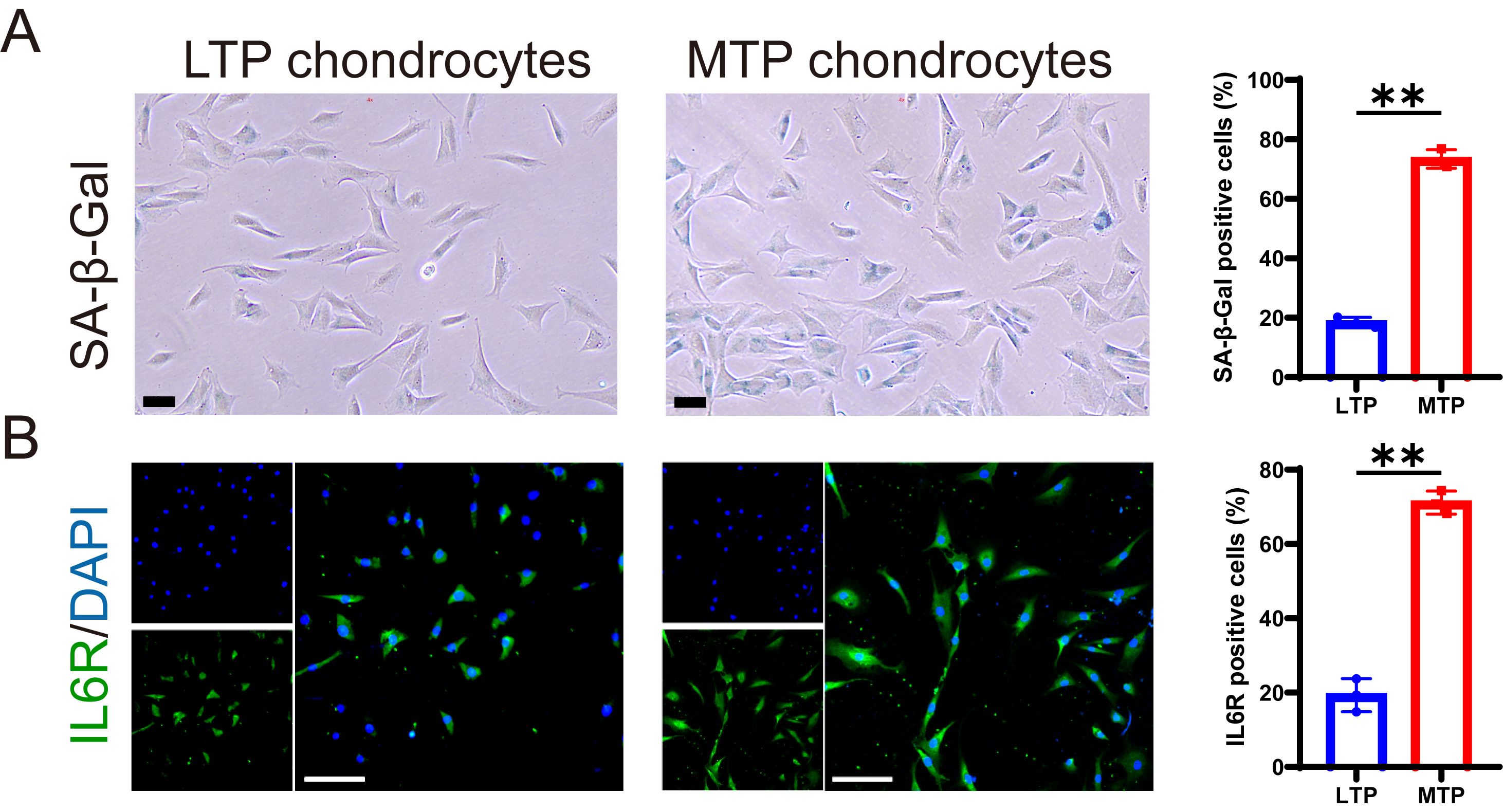


**Supplementary Figure S1.** Representative images showing the SA-β-Gal positivity and IL6R expression levels in LTP or MTP chondrocytes from OA patients’ knee articular cartilage. Scale bar: 100µm. Quantitative data are shown as mean ± SD. A two-sided paired Student's t-test was used for statistical analysis(n=3). **p<0.01.


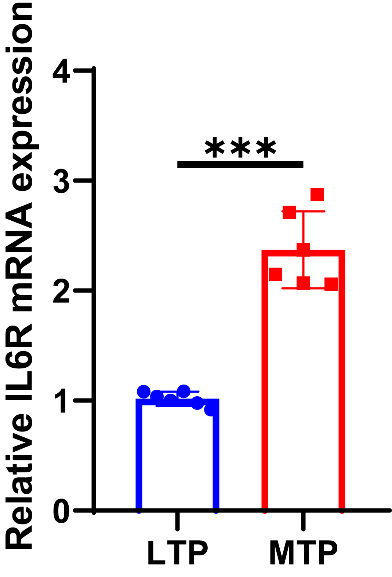


**Supplementary Figure S2.** RT-qPCR analysis was performed to measure IL-6R mRNA levels in LTP and MTP cartilage tissues (n=6). Quantitative data are shown as mean ± SD.***p<0.001.


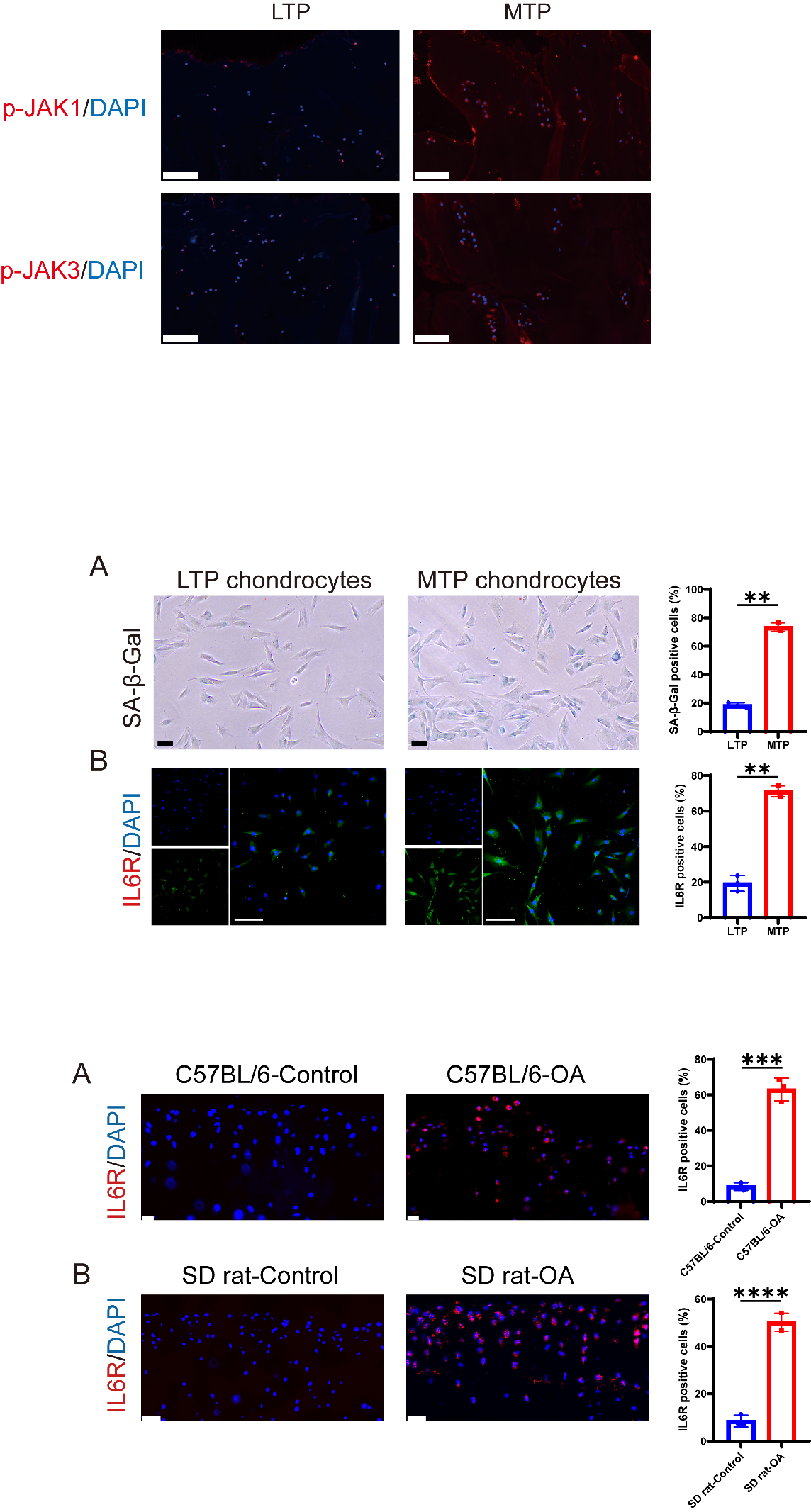


**Supplementary Figure S3.** IL-6R expression was markedly elevated in the cartilage of rat and mice OA models. (A) Immunofluorescence analysis of IL-6R in murine knee cartilage post-4-week DMM or sham surgery, quantifying the percentage of IL-6R-positive cells (n=6). Scale bar: 20 µm. (B) Immunofluorescence analysis was employed to evaluate the expression of IL-6R in rat knee cartilage following 4-week ACLT+DMM or sham surgery, with quantification of the proportion of IL-6R-positive cells (n=6). Scale bar: 20 µm. Quantitative data are shown as mean ± SD. A two-sided unpaired Student's t-test was used for statistical analysis. ***p<0.001, ****p<0.0001.


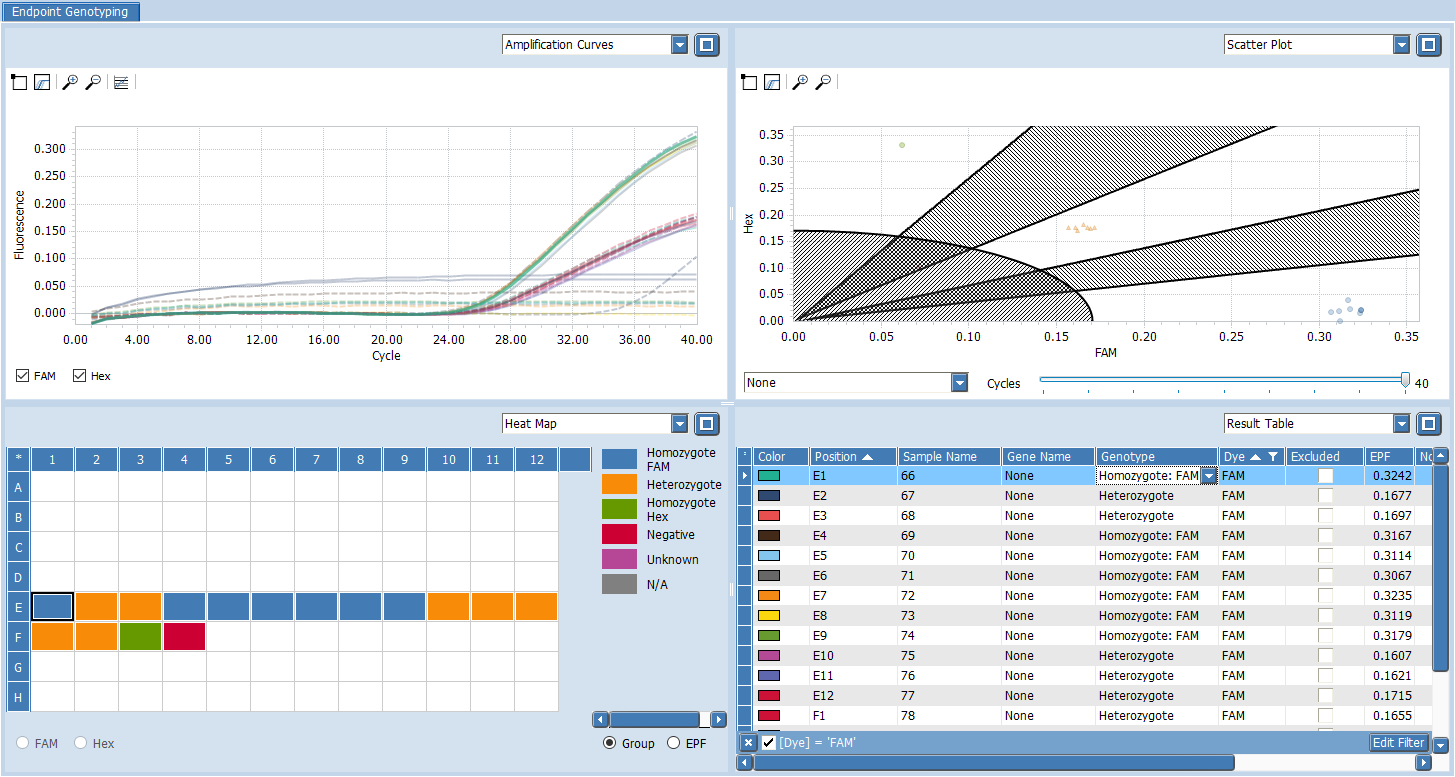


**Supplementary Figure S4.** PCR genotyping of gp130(Y757F) mice.


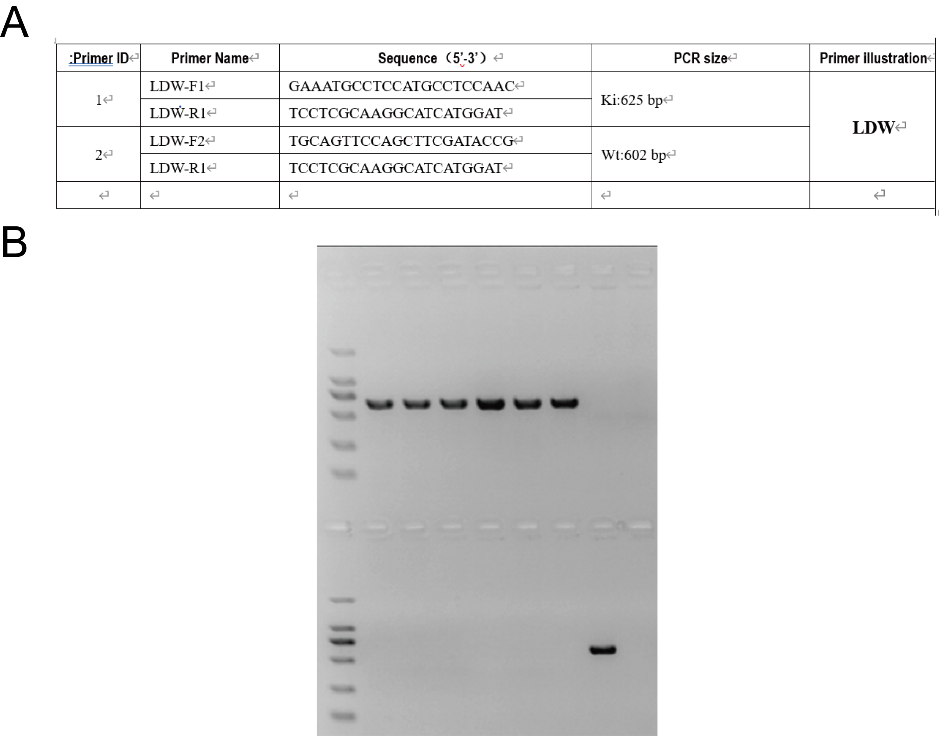


**Supplementary Figure S5.** PCR genotyping of IL-6R gene knockout mice (A, B). B6: negative control, which is the genomic DNA of C57BL/6 mice; N: Blank control, no template control; DL2000 marker: 2000bp\1000bp\750bp\500bp\250bp\100bp.


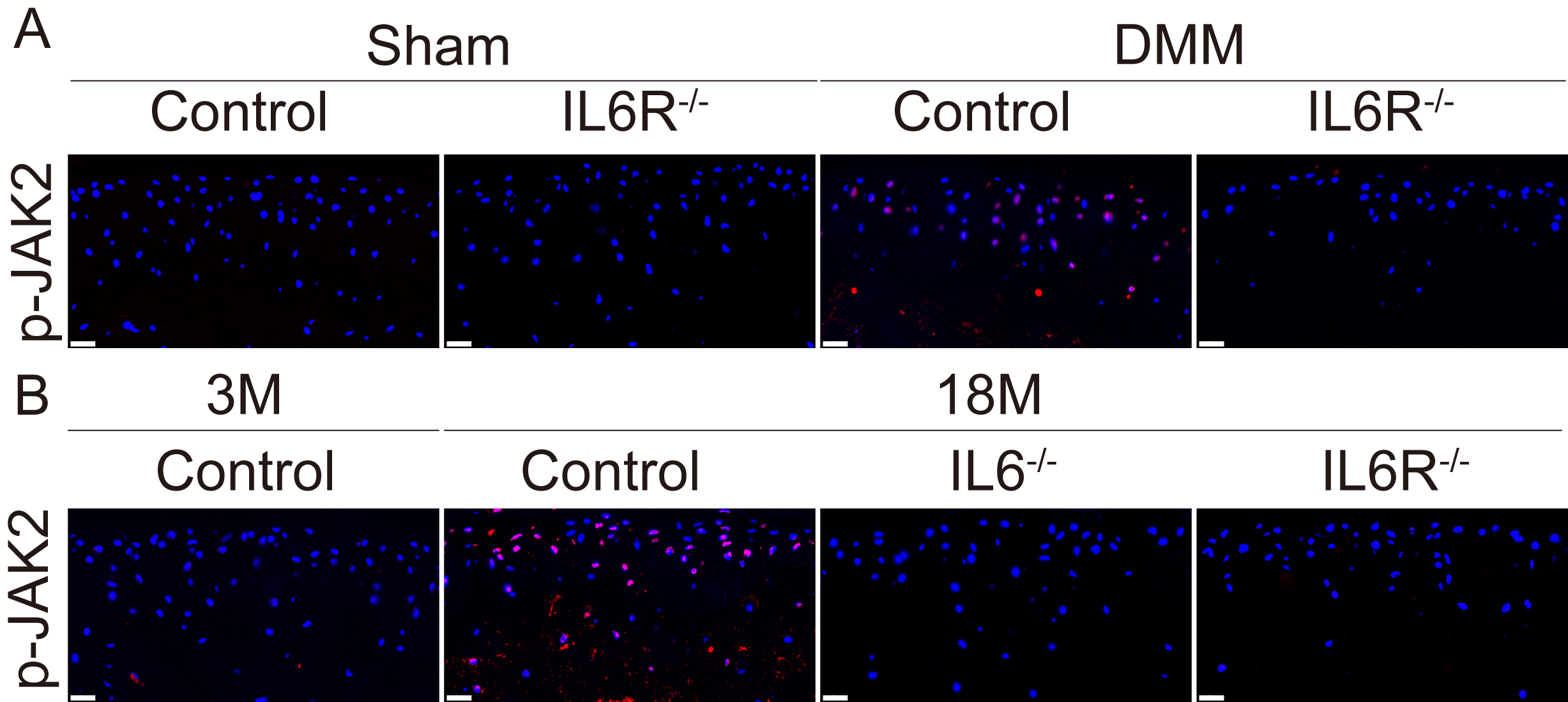


**Supplementary Figure S6.** IL-6R gene knockout reduces p-JAK2 expression in mice with OA induced by DMM surgery or aged mice. Immunofluorescence staining was conducted to evaluate p-JAK2 expression in mouse knee cartilage, with nuclei counterstained using DAPI. Scale bar: 20 µm.


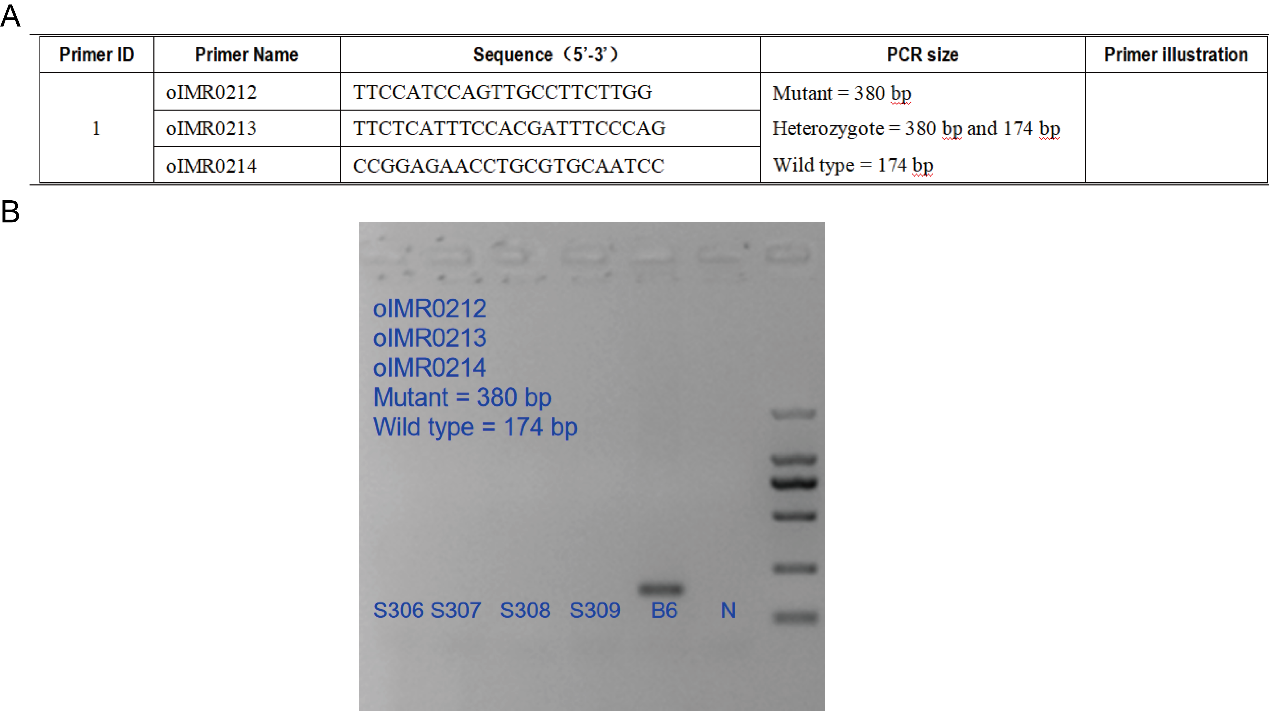


**Supplementary Figure S7.** PCR genotyping of IL-6 gene knockout mice (A, B). B6: negative control, which is the genomic DNA of C57BL/6 mice; N: Blank control, no template control; DL2000 marker: 2000bp\1000bp\750bp\500bp\250bp\100bp.


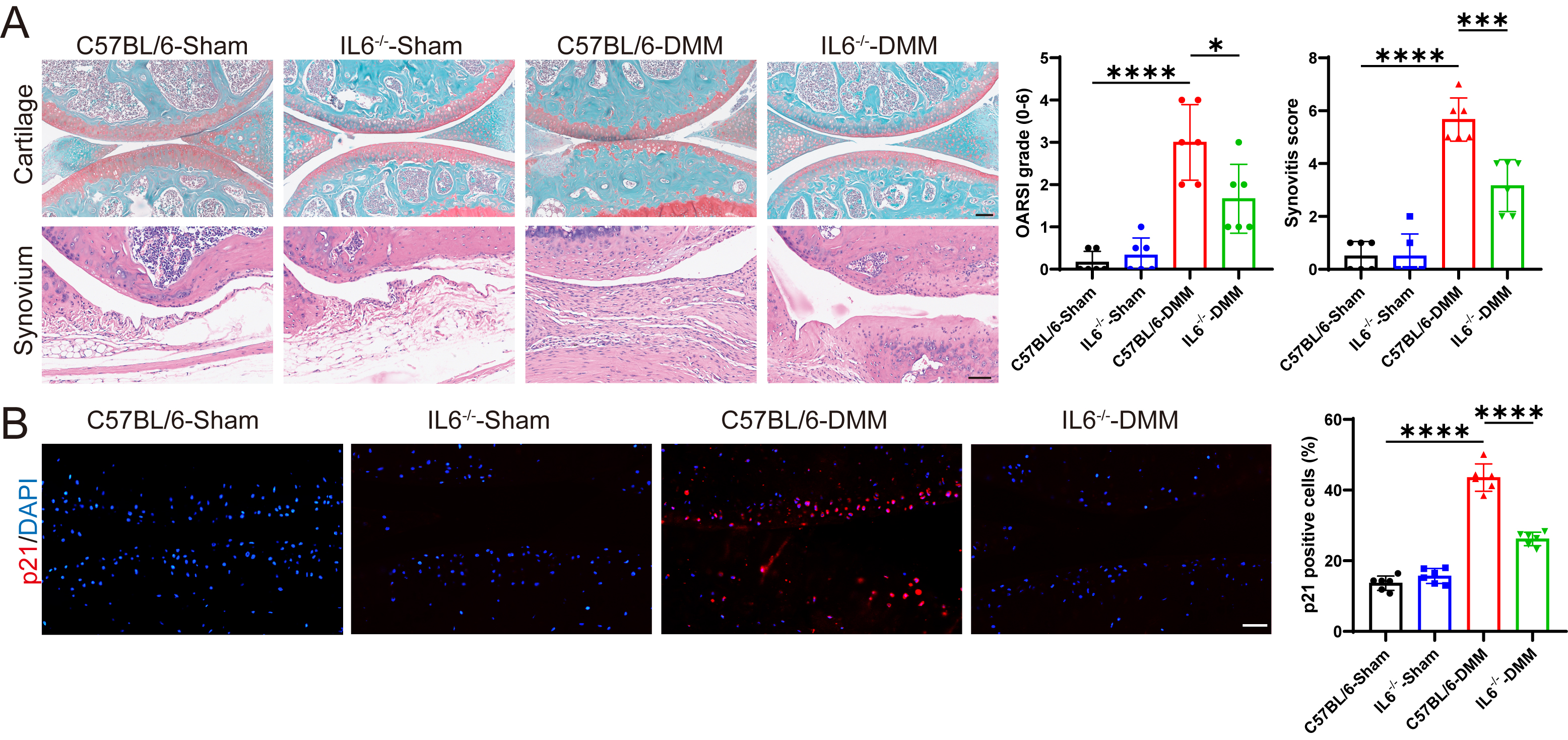


**Supplementary Figure S8.** Knockout of IL-6 alleviates OA and chondrocyte senescence. (A) Representative images of Safranin-O/fast green staining of cartilage tissues and H&E staining of synovium tissues from the indicated groups (C57BL/6 and IL6^-/-^ mice undergoing sham or DMM surgery). OARSI and synovitis scores quantification(n=6). Scale bar: 100µm. (B) Representative images showing the p21 expression levels of cartilage tissues from the indicated groups (C57BL/6 and IL6^-/-^ mice undergoing sham or DMM surgery) and the percentage of p21 positively stained cells in the cartilage were quantified(n=6). Scale bar: 100 µm. Quantitative data are shown as mean ± S.D. One-way ANOVA with Tukey’s multiple comparisons was used for statistical analysis. *p<0.05, ***p<0.001, ****p<0.0001.


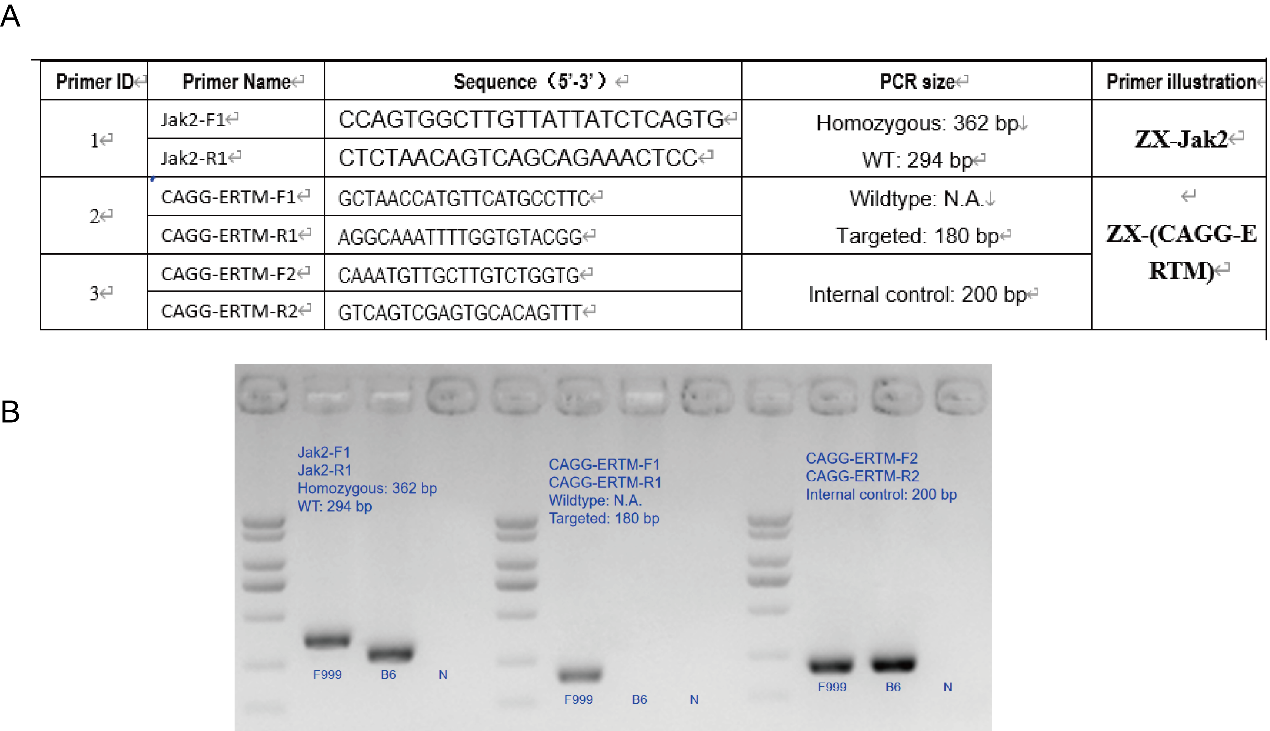


**Supplementary Figure S9.** PCR genotyping of JAK2 gene knockout mice (A, B). B6: negative control, which is the genomic DNA of C57BL/6 mice; N: Blank control, no template control; DL2000 marker: 2000bp\1000bp\750bp\500bp\250bp\100bp.


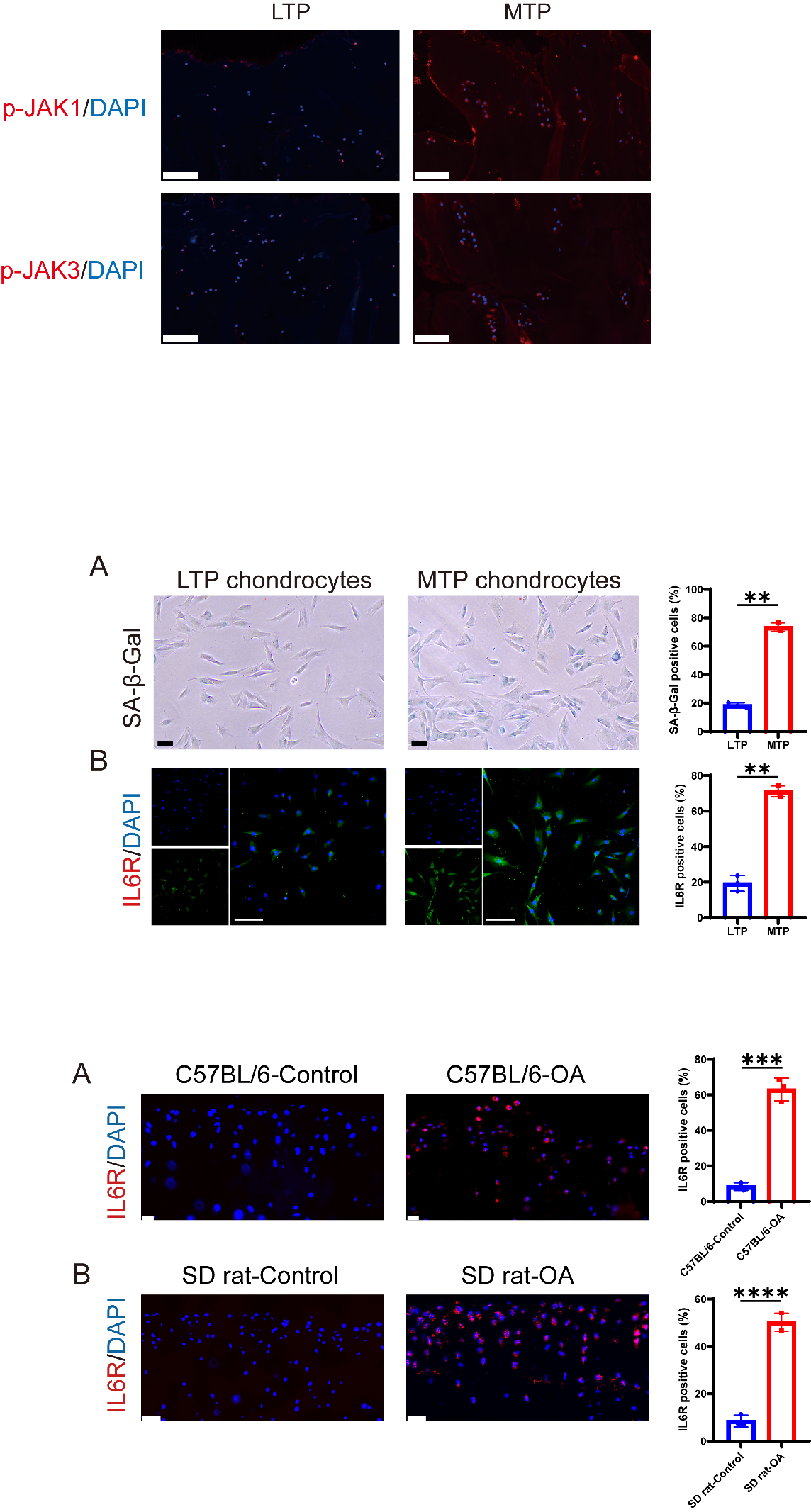


**Supplementary Figure S10.** p-JAK1 and p-JAK3 expression are up-regulation in human OA degenerated cartilage. Immunofluorescence detection of p-JAK1 and p-JAK3 in LTP or MTP cartilage. Scale bar: 100 µm.

**
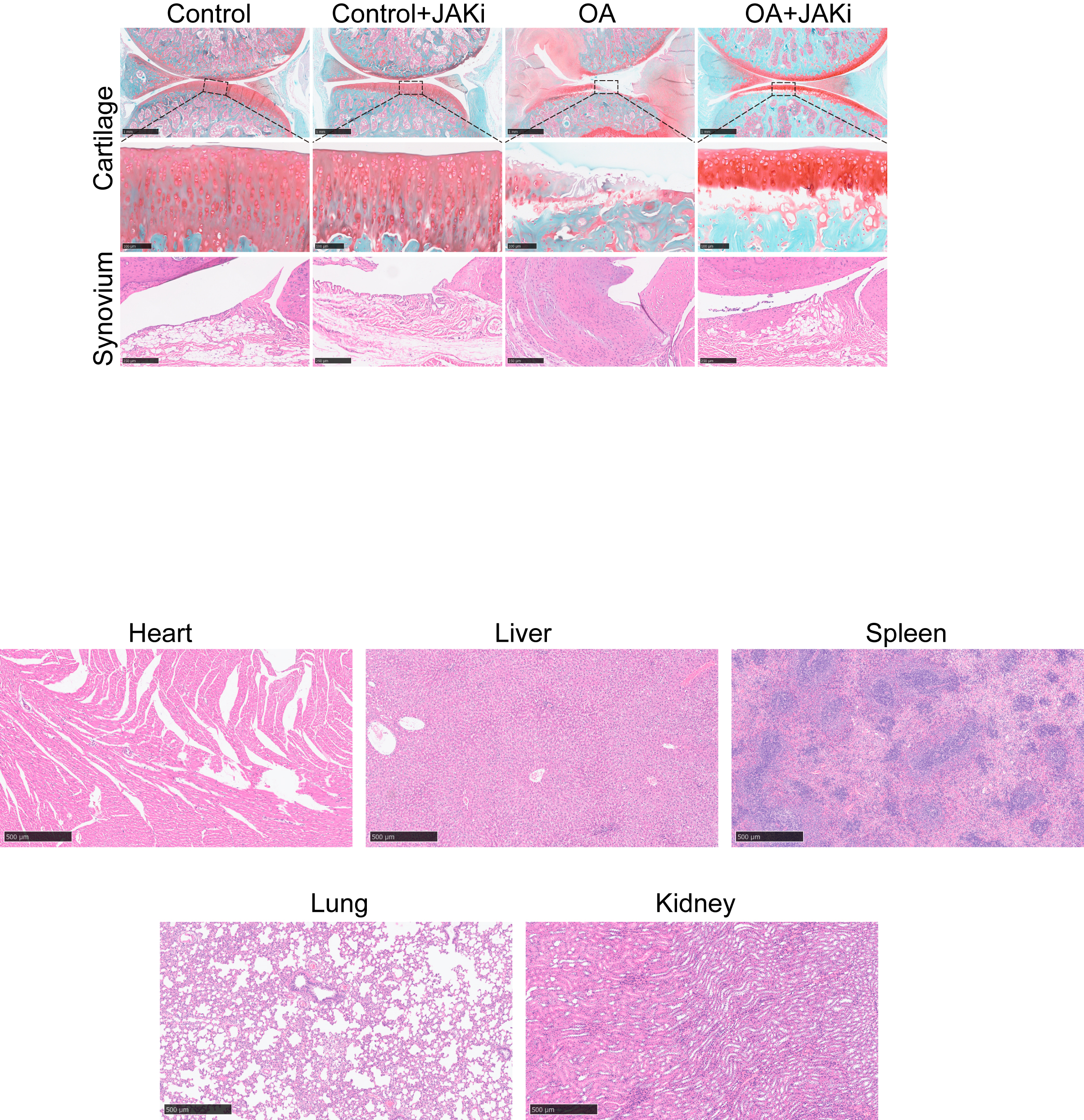
**

**Supplementary Figure S11.** JAKi treatment alleviates OA in rats induced by ACLT+DMM surgery. Cartilage damage and synovitis were evaluated at 8 weeks using Safranin O-Fast Green (SO&FG) and H&E staining. Scale bar: top:1 mm, middle: 100 µm, bottom: 250 µm.


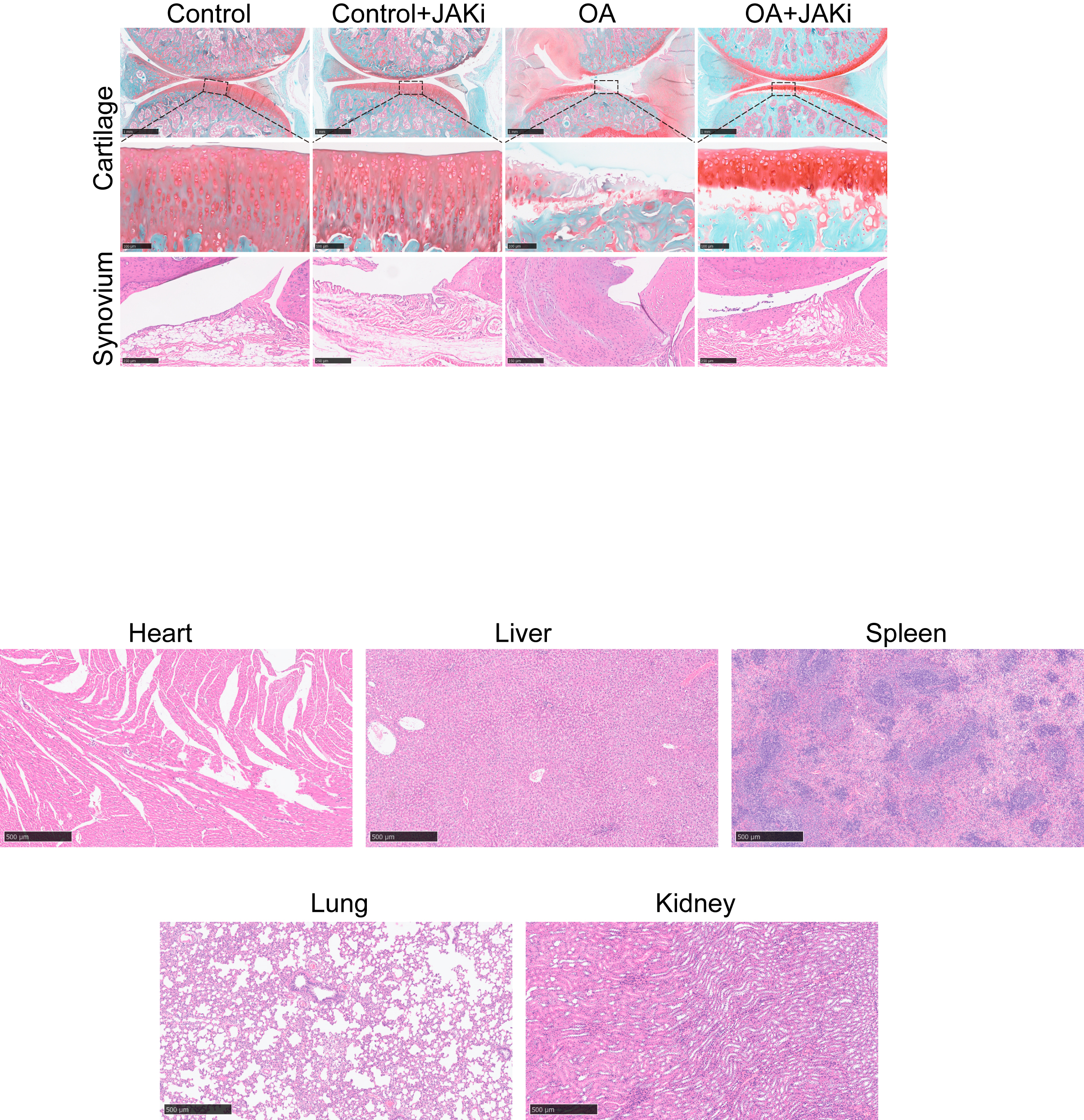


**Supplementary Figure S12**. Safety profile assessment of JAK inhibitors. Representative H&E-stained images of major organs collected after 8 weeks of treatment, highlighting potential histopathological changes.


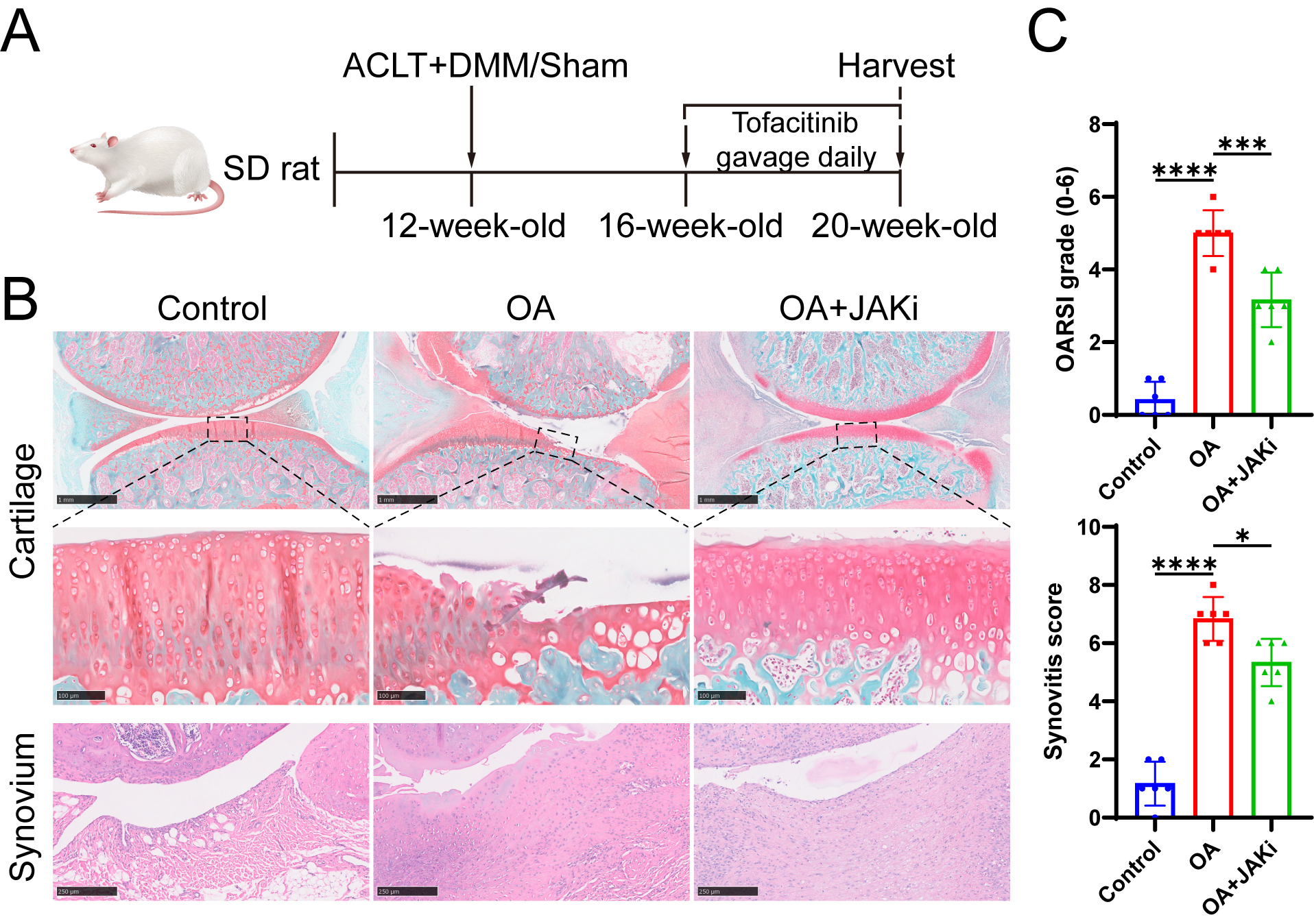


**Supplementary Figure S13.** Delayed administration of JAKi mitigates OA in rats following ACLT+DMM surgery. (A) Schema illustrating the evaluation of delayed administration of JAKi mitigates OA in rats following ACLT+DMM surgery. Rats were administered either vehicle or JAKi (tofacitinib) via gavage daily, beginning 30 days post-surgery, for a duration of 4 weeks. (B) Cartilage damage and synovitis were evaluated at 8 weeks using Safranin-O/fast green and H&E staining. (n=6 rat/group). Scale bar: 1 mm. (C) Quantification of OARSI and synovitis scores. Quantitative data are shown as mean ± SD. One-way ANOVA with Tukey's multiple comparisons was used for statistical analysis. *p<0.05, ***p<0.001, ****p<0.0001.


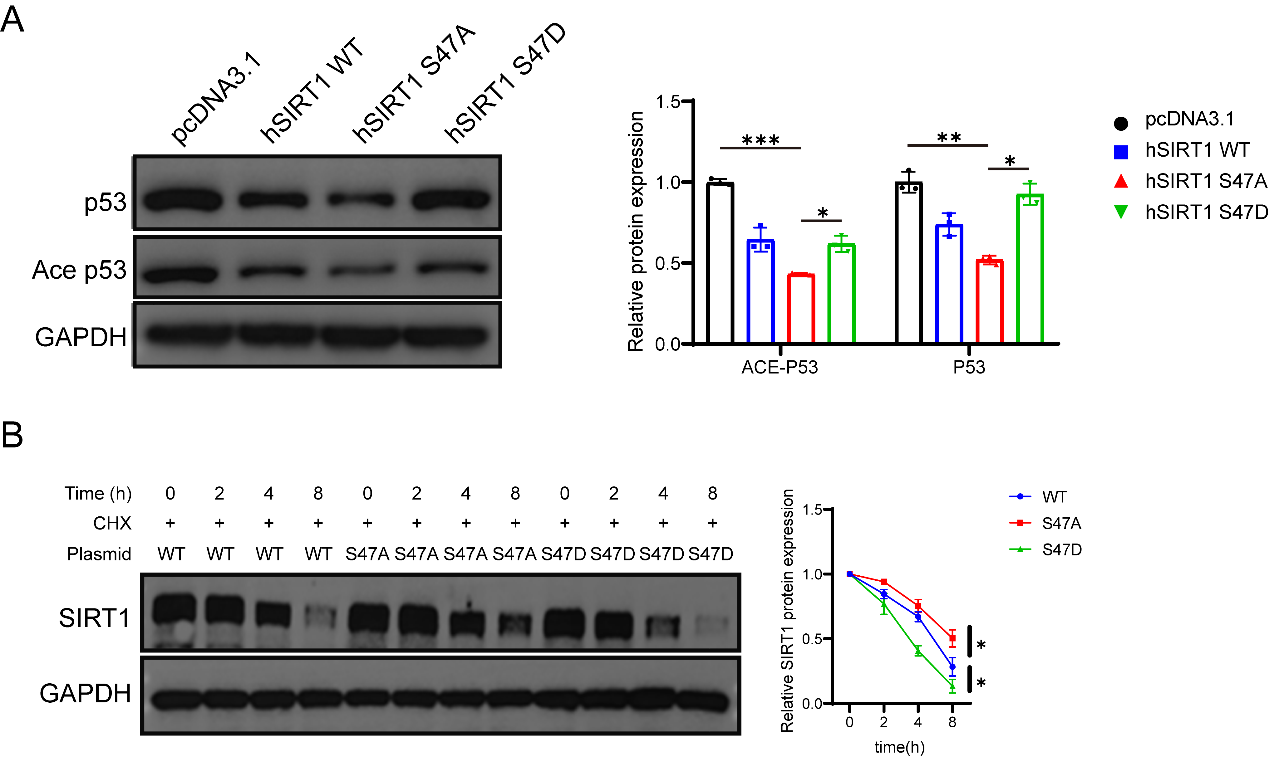


**Supplementary Figure S14.** (A) 293T cells were transiently transfected with pcDNA 3.1 or three other vectors to overexpress human SIRT1 (hSIRT1), the S47A mutant (hSIRT1-S47A), and the S47D mutant (hSIRT1-S47D). Western blotting measured the amount of acetylated p53 48 hours after transfection. The results were presented as the ratio of acetylated p53 to GAPDH. (B) The half-lives of hSIRT1, hSIRT1-S47A, and hSIRT1-S47D were compared to assess the stability of the SIRT1 protein. Protein synthesis was inhibited using cycloheximide (CHX). The abundance of the SIRT1 protein was quantified. Quantitative data are shown as mean ± SD. One-way ANOVA with Tukey's multiple comparisons was used for statistical analysis. *p<0.05, **p<0.01, ***p<0.001.


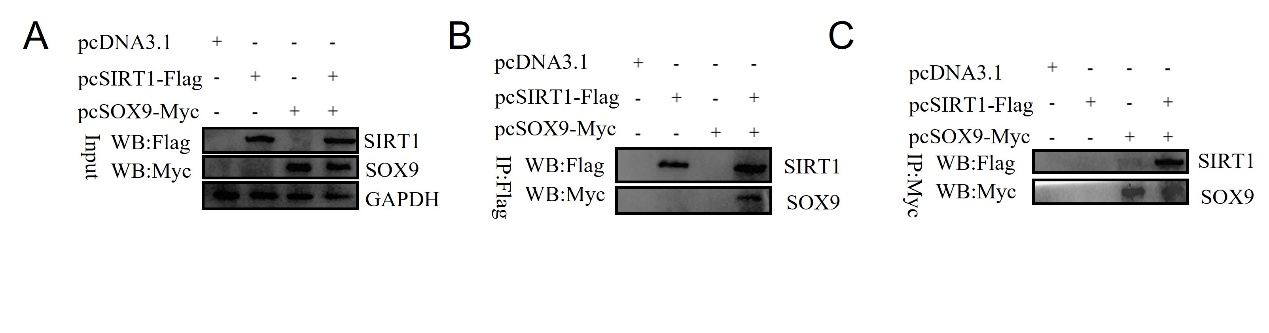


**Supplementary Figure S15.** SIRT1 Interacts with SOX9. (A) Western blotting analysis of SIRT1 and SOX9 overexpression in 293T cells using pcDNA3.1-based plasmids. (B) Co-immunoprecipitation (Co-IP) analysis of the interaction between SIRT1 and SOX9, using Flag antibody for IP. (C) Co-IP analysis of SOX9-SIRT1 interaction, using Myc antibody for IP.


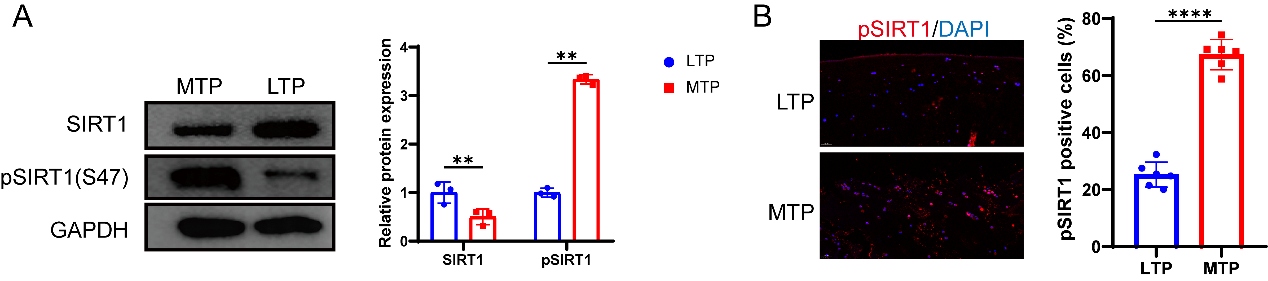


**Supplementary Figure S16.** (A) Western blotting detected and quantified the protein levels of SIRT1 and pSIRT1 in LTP and MTP cartilage blocks (n=3). (B) The expression of pSIRT1 (S47) in chondrocytes was evaluated by immunofluorescence staining in LTP or MTP cartilage (n=6), and the percentage of pSIRT1 (S47) positively stained cells in the cartilage was quantified. Scale bar: 50 µm. Quantitative data are shown as mean ± SD. A two-sided paired Student's t-test was used for statistical analysis. **p<0.01, ****p<0.0001.


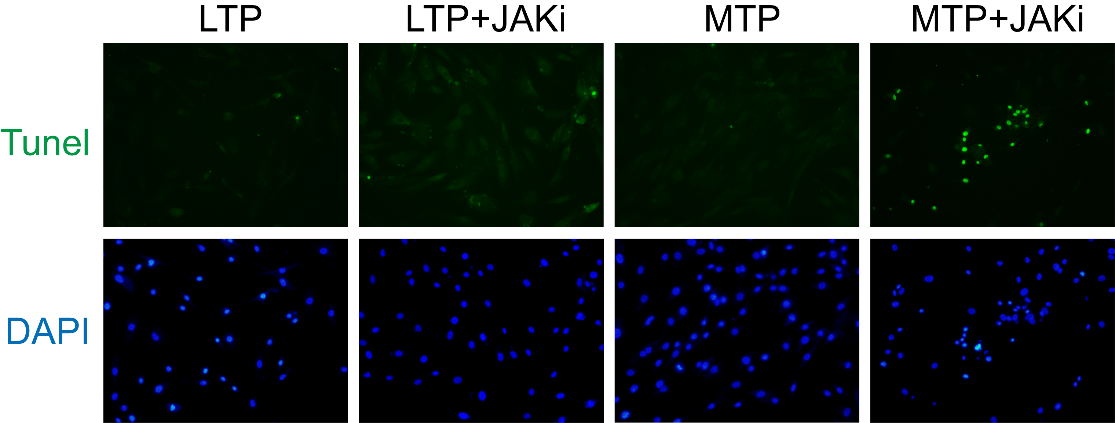


**Supplementary Figure S17.** Representative TUNEL staining images of chondrocytes from LTP or MTP cartilage tissues cultured for 3 days with or without JAKi.


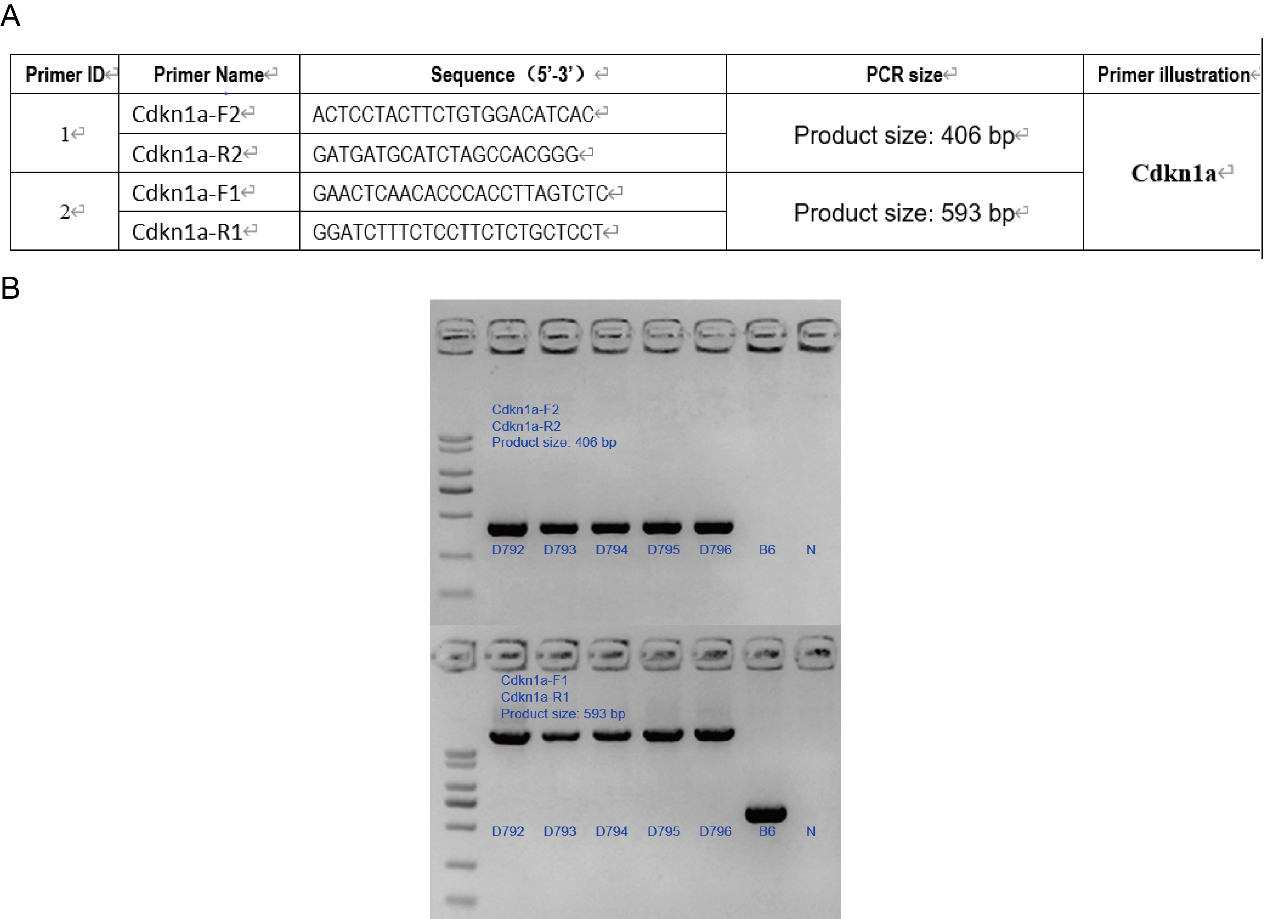


**Supplementary Figure S18.** PCR genotyping of p21-3MR mice (A, B). B6: negative control, which is the genomic DNA of C57BL/6 mice; N: Blank control, no template control; DL2000 marker: 2000bp\1000bp\750bp\500bp\250bp\100bp.
